# Carbon source priority and availability limit bidirectional electron transfer in freshwater mixed culture electrochemically active bacterial biofilms

**DOI:** 10.1186/s40643-023-00685-w

**Published:** 2023-09-20

**Authors:** Karina Michalska, Robert Keith Brown, Uwe Schröder

**Affiliations:** Institute of Biochemistry, University of Greifswald, Felix-Hausdorff-Str. 4, 17489 Greifswald, Germany

**Keywords:** Diauxie, Bioelectrochemistry, Electrochemically active bacteria, Bidirectional in-/direct external electron transfer

## Abstract

This study investigated, if a mixed electroactive bacterial (EAB) culture cultivated heterotrophically at a positive applied potential could be adapted from oxidative to reductive or bidirectional extracellular electron transfer (EET). To this end, a periodic potential reversal regime between − 0.5 and 0.2 V vs. Ag/AgCl was applied. This yielded biofilm detachment and mediated electroautotrophic EET in combination with carbonate, i.e., dissolved CO_2_, as the sole carbon source, whereby the emerged mixed culture (S1) contained previously unknown EAB. Using acetate (S2) as well as a mixture of acetate and carbonate (S3) as the main carbon sources yielded primarily alternating electrogenic organoheterotropic metabolism with the higher maximum oxidation current densities recorded for mixed carbon media, exceeding on average 1 mA cm^−2^. More frequent periodic polarization reversal resulted in the increase of maximum oxidative current densities by about 50% for S2-BES and 80% for S3-BES, in comparison to half-batch polarization. The EAB mixed cultures developed accordingly, with S1 represented by mostly aerobes (84.8%) and being very different in composition to S2 and S3, dominated by anaerobes (96.9 and 96.5%, respectively). S2 and S3 biofilms remained attached to the electrodes. There was only minor evidence of fully reversible bidirectional EET. In conclusion the three triplicates fed with organic and/or inorganic carbon sources demonstrated two forms of diauxie: Firstly, S1-BES showed a preference for the electrode as the electron donor via mediated EET. Secondly, S2-BES and S3-BES showed a preference for acetate as electron donor and c-source, as long as this was available, switching to CO_2_ reduction, when acetate was depleted.

**Graphical Abstract:**

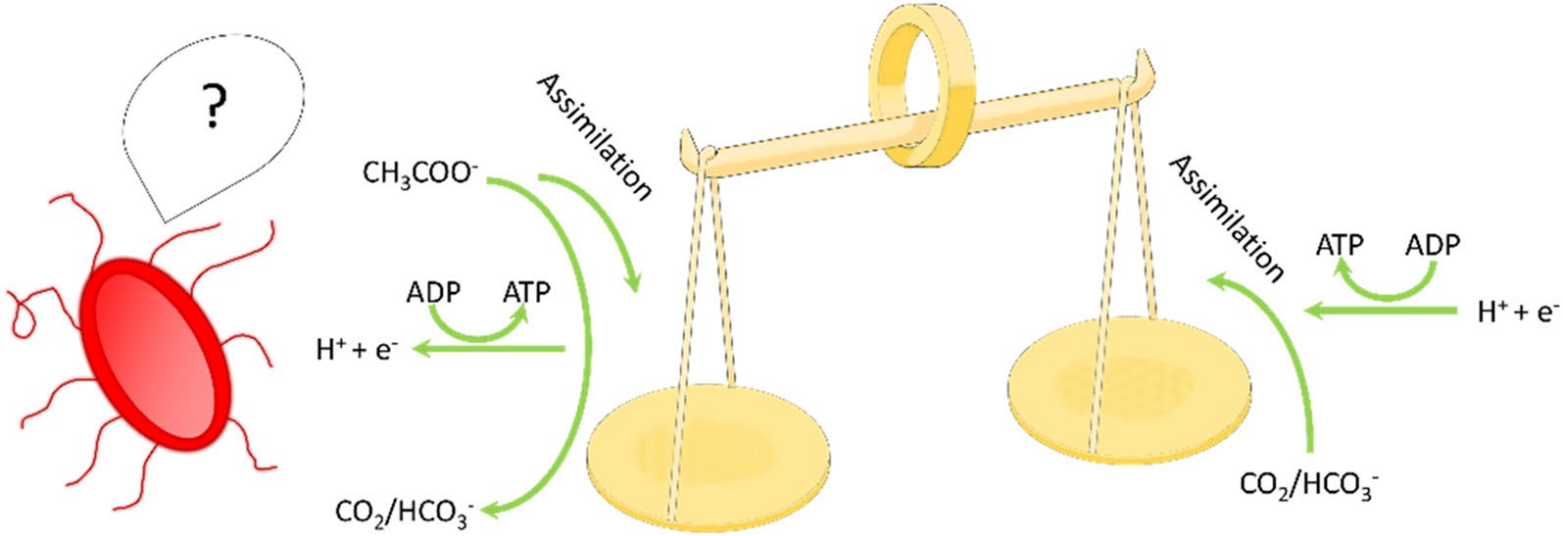

**Supplementary Information:**

The online version contains supplementary material available at 10.1186/s40643-023-00685-w.

## Introduction

Bioelectrochemical systems (BES) or microbial electrochemical technologies utilize microorganisms as biocatalysts (Kracke et al. 2015; Chatterjee et al. 2019; Logan et al. 2019). These living cells, microscopic in size but colossal in terms of their biotechnological potential, are employed for either electricity production from organic compounds, i.e., electrogenesis from organoheterotrophic metabolism transferring electrons from the cell to an electrode (Fricke et al. 2008; Pereira et al. 2022) or reduction of CO_2_/organics into (larger) organic energy storage molecules and/or biomass, i.e., litho- or electroautotrophic metabolism (Vassilev et al. 2018; Jourdin et al. 2020; Cabau-Peinado et al. 2021).

Donating or accepting electrons allows microorganisms to sustain their metabolism, because the electrons flow through the respiratory chains countered by proton flow, which in turn allow e.g., ATP synthesis. Compared to the cell size the external/extracellular electron transfer (EET) can occur over relatively long distances, and its kinetics are controlled by the potential at the electrode (Chong et al. 2018; Riedl et al. 2019; Paquete et al. 2022). Two directions of EET, namely electroautotrophic, i.e., cathodic or reductive, and electrogenic, i.e., anodic or oxidative, have been thoroughly investigate for direct applications, e.g., microbial electrosynthesis (MES) or microbial fuel cells (MFC), respectively (Kumar et al. 2017; Jiang and Zeng 2019; Li et al. 2021). Although substantial progress has been made in the understanding of microbial EET mechanisms, the great majority of performed research are dedicated only to a one-direction, with most research focused on electrogenic pathways/EET, whereas the interest for electroautotrophic pathways/EET has developed only more recently (Rosenbaum et al. 2011; Kumar et al. 2017; Karthikeyan et al. 2019).

Some electrochemically active bacteria (EAB) are able to switch between electrogenic and electroautotrophic EET (Ross et al. 2011; Okamoto et al. 2014; Yu et al. 2015, 2018). This was later further investigated under the term bidirectional EET or canodic (from cathodic + anodic) (Yates et al. 2017; Mickol et al. 2021), which implies the ability of EAB to adapt and rapidly transition between electrogenic and -autotrophic EET (Pous et al. 2016; Yates et al. 2017; Izadi et al. 2021; Xie et al. 2021). This research paved the way for combined bioelectrochemical energy storage (electroautotrophy) and power generation (electrogenesis) in a single device. It could also facilitate better cultivation and control of EAB for bioelectrosynthesis applications.

To explore the potential of bidirectional EET, the fundamental aspects of the underlying molecular mechanism and relevant storage compounds need to be fully understood. A bottleneck for such research, however, is the time-consuming and sensitive enrichment step. Polarization reversal is a method initially developed to control pH in the cathode of a microbial electrolysis cell (MEC) for enhancing hydrogen production (Yang et al. 2017). With time it has been recognized as a fast and effective way for biocathode enrichment and promoting the switch from electrogenesis to electroautotrophic activity (Hartline and Call 2016; Liang et al. 2023).

Several different approaches have been proposed for the formation of a canodic biofilm. The commonly applied strategies involve so far either fully autotrophic cultivation combined with repeatedly polarization reversal (Yates et al. 2017) or heterotrophic cultivation (anodic pre-enrichment) followed by autotrophic polarization switch (Izadi et al. 2021). Switching to autotrophic and cathodic operation after initial heterotrophic cultivation, however, is not always successful for developing carbon dioxide reducing biocathodes (Saheb-Alam et al. 2018). A common feature of both strategies is maintaining/forcing autotrophic metabolism through—periodic—polarization reversal, i.e., removal of all organic carbon sources prior to the first switching (Pous et al. 2016; Izadi et al. 2021). That in turn leads to establishing bidirectional EET mainly through chemolitoautotrophic microorganisms, whose growth is favored under such conditions, whereas the question of the involvement of organotrophic community in bidirectional EET still remains open.

A few pure heterotrophic electroactive cultures are known to date to be capable of performing bidirectional EET, e.g., *Shewanella oneidensis* (Yong et al. 2014; Zou et al. 2019; Ikeda et al. 2021; Li et al. 2021) and *Geobacter* sp. (Soussan et al. 2013). Bidirectional EET in mixed culture biofilms has yet to be deeply investigated. Taking into account that most of EABs are recognized as heterotrophic bacteria without ability to fix carbon dioxide (Rabaey and Rozendal 2010), ignoring their usefulness for bidirectional biofilm studies should be considered a great negligence.

Based on the above, bidirectional EET was investigated in mixed EAB cultures, grown heterotrophically under an applied potential, which favors electrogenic activity. This was followed by switching to a more negative potential. Also, this was done in the presence of organic, inorganic carbon or a mixture thereof. This was done to see, if fast switching is possible with respect to biofilm stability, as well as to determine, in particular, if CO_2_/CO_3_ available after acetate oxidation would trigger reductive activity. To round out the electrochemical and HPLC measurements, the composition of the microbial community in the BES was determined to evaluate the adaptation and differentiation of EAB within the experiments.

## Materials and methods

If not stated otherwise all chemicals were from Sigma/Merck, Carl-Roth Germany and of analytical grade or equivalent purity.

To establish a stable and reliable biofilm in relatively short time frame, a heterotrophic growth strategy has been applied. A mixed anodic culture  was inoculated into the BES (see "BES-operation and electrochemical analyses" section) and cultivated in 10 mM acetate and 30 mM carbonate medium over the period of several weeks. C-source amounts were based on those used by (Izadi et al. 2021). This cultivation was performed at constant anodic potential (Phase 1, Table 1). When biofilm formation was confirmed, the BES were organized into 3 independent triplicates groups: S1, S2 and S3, each fed with a media containing different C-sources (see Table 1), and the applied potential was switched to promote the development of bidirectional electron transfer. Adaptation of EAB biofilm to polarization switching as well as the resulting biofilm composition and behavior in relation to the initial available C-sources were investigated in two stages, first with a half-batch polarization reversal (HBPR) followed by a periodic polarization reversal (PPR) (Phases II and III, respectively, in Table 1).

**Table 1:** An overview of the research methodology

Phase I	CULTIVATION		
Carbon source	NaHCO_3_ + CH_3_COOH		
Procedure	CA (0.2 V)		
Phase II	HBPR – half-batch polarization reversal phase		
Reactor	S1-BES	S2-BES	S3-BES
Carbon source	NaHCO_3_	CH_3_COOH	NaHCO_3_ + CH_3_COOH
Procedures	CA (− 0.5 V) → CV (− 0.5 V to + 0.2 V) → CV with OC controls → CA (+ 0.2 V) → CV (+ 0.2 V to -0.5 V) → CV with OC controls		
Phase III	PPR – periodic polarization reversal phase		
Reactor	S1-BES	S2-BES	S3-BES
Carbon source	NaHCO_3_	CH_3_COOH	NaHCO_3_ + CH_3_COOH
Procedures	CA (− 0.5 V) → CA (0.2 V)	CA (0.2 V) → CA (− 0.5 V)	CA (0.2 V) → CA (− 0.5 V)

*CA* chronoamperometry—application of a constant potential, *CV* cyclic voltammetry: application of a periodically changing potential. *OC* open circuit, i.e., electrodes are suspended in media, but not connected to the potentiostat, except for short periods

### BES setup

All bioelectrochemical experiments were performed with biological triplicates in dual-chamber glass reactors made from 250 mL bottles (SCHOTT, Germany), into which round 50 mm diameter borosilicate flat glass flanges (Rettberg, Germany) were added, creating an H-cell. The flange increased the nominal volume to approx. 280 mL per chamber. The chambers were separated by a cation exchange membrane (Fumapem, FKE-PP-75, Fumatech, Germany). Graphite disks with a projected surface area of 3.14 and 7.07 cm^2^ were used as working (WE) and counter electrode (CE), respectively. Ag/AgCl reference electrodes (saturated KCl, Sensortechnik Meinsberg, Germany, 0.197 V vs. SHE) were used and if not otherwise stated all potentials are given vs. this reference. Additionally, graphite rods with a projected surface area of 2.9 cm^2^ were used as a secondary WE. These were only tested periodically and otherwise not connected to a potentiostat, i.e., they were used as open circuit (OC) controls. All measurements were performed at a temperature of 35 ± 1 °C and under potentiostatic control (VMP3, BioLogic, France).

### BES-operation and electrochemical analyses

250 mL of growth medium were used in the working electrode chamber and were replenished after each batch. The base medium consisted of 12.5 mL L^−1^ of trace element solution and 12.5 mL L^−1^ of vitamin solution (Balch et al. 1979) in 50 mM phosphate buffer solution (PBS) as described previously (Baudler et al. 2014) with the addition of 1.19 g L^−1^ NH_4_Cl. Initially, a mixture of 0.82 g L^−1^ ≈ 10 mM of NaAc + 2.50 g L^−1^ ≈ 30 mM of NaHCO_3_ was used as the carbon sources. Hereafter, three BES were fed with either 2.50 g L^−1^ of carbonate (S1-BES), 0.82 g L^−1^ of acetate (S2-BES) or mixed 0.82 g L^−1^ of acetate + 2.50 g L^−1^ of carbonate (S3-BES). The solution for the counter electrode chamber consisted of PBS with 1.19 g L^−1^ NH_4_Cl and 12.5 mL L^−1^ of mineral solution. All final solutions/media were purged with nitrogen for at least 30 min before use to ensure the system started under anaerobic conditions. Also, small amounts of ethanol were present in the media solutions (verified in zero-samples), originating from the 70% ethanol solution used for cleaning the reusable metal cannulas etc. The ethanol concentration amounted to 1.0 ± 1.1 mM during HBPR and 0.5 ± 0.5 mM during PPR.

24-h prior to inoculation, an experiment was conducted to obtain baseline electrochemical data for the BES. This was done by applying chronoamperometry (CA) and cyclic voltammetry (CV) in a repeating loop with BES filled with media, but without being inoculated. The potential variations of WE were as follows: starting with CA at + 0.2 V for 3h, then for CV at potential ranging from + 0.2 V to − 0.5 V, ending at -0.5 V, with a scan rate of 1mV s^−1^ for 4 cycles, followed by CA with a potential of -0.5 V for 3h and the CV with potential range from − 0.5 V to + 0.2 V, ending at 0.2 V, with a scan rate of 1mV s^−1^ for 4 cycles.

**Fig. 1:**
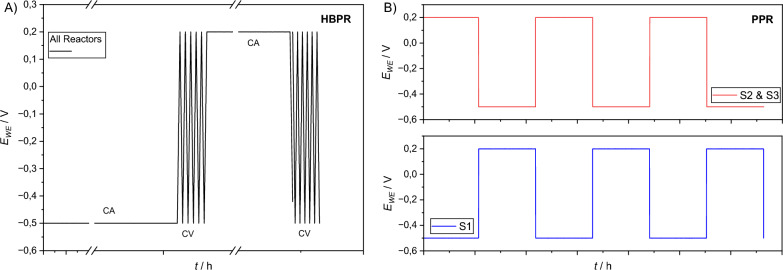
Graphic illustration of applied potential switch over the course of single batch: **A** *HBPR* half-batch polarization reversal phase. **B** *PPR* periodic polarization reversal phase. Both (**A**) and (**B**) have the same time scales of 7 d

For inoculation, 15 mL of the growth medium was used to suspend mixed EAB biofilm/biomass harvested from a long-term operating BES-mother reactor fed only on 10 mM acetate (but otherwise the same PBS, vitamin and mineral solutions). All 9 BES were polarized at + 0.2 V. A single batch lasted 7 days for all experimental stages and cultivation was done for 6 batches. After cultivation, the 9 BES were split into three (S1, S2 and S3) triplicates and were operated under half-batch polarization reversal. A combined (longer CA and shorter CV periods) approach was chosen to investigate, as described in “Introduction” section, the effect of polarization reversal over a prolonged period of time in combination with different C-sources. This started with nominal cathodic polarization at − 0.5 V for 81 h followed by CV during which the potential was scanned in range from − 0.5 V to + 0.2 V ending at + 0.2 V with the scan rate of 1 mV s^−1^ for 4 cycles. Halfway through the batch (half-batch polarization reversal—HBPR) the polarization of the WE was switched to nominal anodic polarization (+ 0.2 V) for 81 h. This was followed by CV ranging from + 0.2 V to − 0.5 V ending at − 0.5 V with a scan rate of 1 mV s^−1^ for 4 cycles. The polarization procedure is illustrated in Fig. 1a. Liquid samples were taken prior to the CV operations. After completion of each CV-Step of the procedure, the OC control WE (see "BES setup" section) were connected and CV measurements with the same settings were done with them. CV-Steps and CVs with OC controls as well as sampling took up the remaining 3 h of each half batch. This was repeated for 5 batch-cycles.

For periodic polarization reversal – PPR – S1-BES were started at cathodic polarization, followed by corresponding CV and then switching, while S2-BES and S3-BES were started at anodic CA polarization. This is shown in Fig. 1b. The first CA was followed by switch in polarization to the other applied potential, i.e., − 0.5 to + 0.2 or + 0.2 to − 0.5 V. The BES were operated without CV operations in between the CA and periods of anodic and cathodic polarization were shorter, i.e., a total of three periods each 27.8h long. This was repeated for 8 batches with the addition that the concentration of carbon sources was decreased by a half for the sixth batch and onwards for the PPR experiments.

### Chemical analyses

To investigate the changes in media composition in relation to the applied electrochemical conditions, medium samples were regularly collected from the BES for further analysis. Samples were taken from fresh media, at the end of each batch from each BES and after half of each HBPR batch. The COD of samples before and after each cycle were measured using cuvette tests (LCK 514 and LCK 314, Hach Lange, Germany) in a spectral photometer (DR3900, Hach Lange, Germany). The pH of the medium was measured at the beginning and the end of each batch (pH-Meter, Hanna Instruments, USA).

High performance liquid chromatography (1260 Infinity II chromatograph, Agilent Technologies, USA) was used to measure the concentrations of both organic oxidation and reduction products (e.g., succinate, acetate, propionate, butyrate, ethanol) in all samples. The device was equipped with an Aminex HPX-87H column (Bio-Rad Laboratories, USA), a refraction index detector (RI), and a diode array detector (DAD). The measurements were performed under the following conditions: column temperature 60 °C, eluent 0.0025 M H_2_SO4, flow rate 0.6 mL min^−1^.

### Microbial community analysis

Microbial community analysis was performed on both biofilm and the liquid samples taken from the BES at the end of the experiments, from the PBS used to prepare these samples and all media as well as from the BES-mother reactor used to inoculate these BES. Biofilms/biomass was collected from each WE and in some S1-BES from the wall of the WE chamber by scraping it off with a sterile spatula. The biomass was transferred into fresh phosphate buffer and sealed in falcon tube. 15 mL of well-mixed (depleted) medium from each BES was taken as well. Microbial community analysis (MCA) was done by AMODIA Bioservice GmbH, Braunschweig, Germany. Total microbial DNA was isolated from the samples, and was used for a universal amplification of a 16S rDNA fragment for the detection of bacteria. The amplified PCR products were separated using single strand conformation polymorphism (SSCP) method. For every sample, a bacterial DNA profile was generated. Each band in the profile represented a specific DNA sequence and was assigned to a bacterial species. For species identification, the bands were isolated and analyzed using DNA sequencing and database inquiry. The alignment with characterized sequences in the database allowed the identification of the next homologous sequence and the respective bacterial species.

## 3. Results and discussion

### Biofilm cultivation

**Fig. 2:**
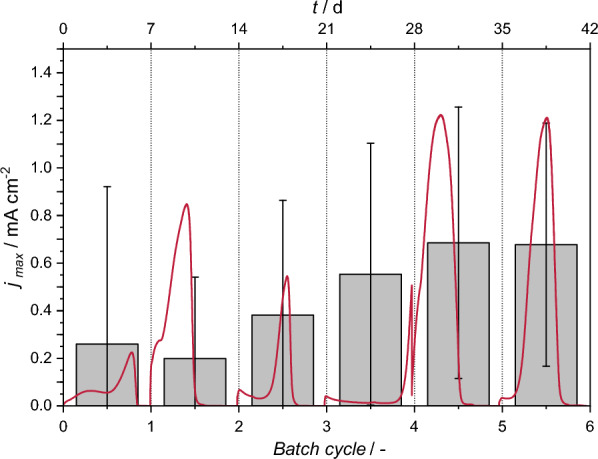
The progression of maximum current densities (columns) and their fluctuations expressed as SD (error bars) over the course of cultivation phase for tested BES. Red lline shows, exemplarly, the actual course of current density development of one BES

Heterotrophic, i.e., anodic, biofilm formation occurred in all BES during the first batch cycle. First peaks are characterized with rather low average maximum current densities on average up to 0.25 mA cm^−2^. Significant differences between all replicates were observed in terms of the values of maximum current densities (see Fig. 2) as well as in terms of turnover duration. The best performance was observed in last 2 cycles, with 3 out of 9 BES exceeding a *j*_*max*_ of 1.2 mA cm^−2^.

Acetate was increasingly consumed in the course of the cultivation along with the slow diminishing of the observed fluctuations in current densities. Stationary conditions were reached after 6 batches (42d) with reproduceable anodic current generation, with respect to each replicate and to the average of all the BES, and full consumption of acetate. The maximum average current density calculated for all BES (≈ 0.70 mA cm^−2^) was obtained after 45 h in the last two cycles. COD removal at the end of the cultivation stage in majority of the BES exceeded 80%; with 2 BES at about 10% lower.

To distribute the activity of formed biofilms uniformly and thus ensure possible equal/comparable initial conditions over the course of the experiment for HBPR and PPR stages, the BES were grouped into 3 sets, each triplicate consisting of one with higher, one mid and one lower performing BES. The three groups of triplicate BES were then used for bidirectional EET experiments and fed with different carbon sources.

### Half-batch polarization reversal

Half-batch polarization reversal (HBPR) started after cultivation was complete. The first group of BES was fed with the medium containing carbonate, the second with acetate and the third with a 50/50-mixture of carbon sources. These are designated S1, S2, and S3, respectively. See Table 1 and  "BES-operation and electrochemical analyses" section for details on the procedure.

**Fig. 3:**
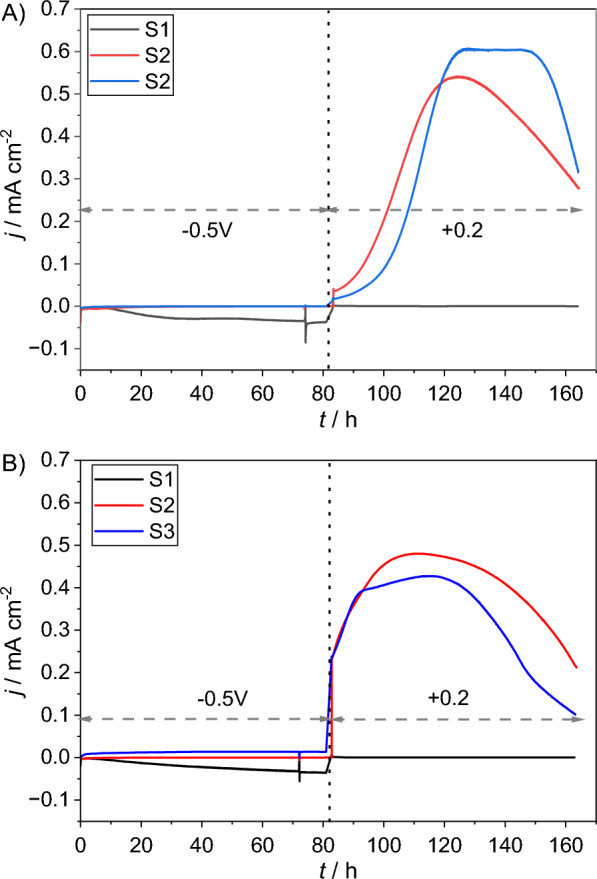
Average current densities over the course of the first batch of HBPR in relation to the carbon source and at the respective applied potrential in the **A** 1st and **B** 4th batch. Sharp verticle lines indicate when CV was carried out

The different carbon sources provided to the BES influenced their behavior significantly. Figure 3 depicts the resulting average current densities over the course of the 1st and 4th batches in relation to the carbon source. Reductive (i.e., negative) current was produced only by the BES fed with carbonate (Additional file 1: Fig. S1) at − 0.5 V, whereas for acetate and mixed carbon media (Additional file 1: Fig. S2, S3) no reductive current was observed. Conversely no oxidative (i.e., positive) current was observed in the S1-BES at + 0.2 V. These observations were consistent for all the batches in HBPR. Based on these results and within the given range of polarization, it would seem that substrate type or carbon source is the deciding factor on whether or not oxidative or reductive current is observed.

Interestingly, one of the S3-BES began to evolve oxidative current during polarization at − 0.5 V beginning with batch 3 (example shown in Additional file 1: Fig. S1). This was not the reactor that reached the highest maximum current density at the end of the cultivation, then only reaching 0.45 mA cm^−2^. We hypothesize that due to the limited amount of alternative (SO_4_^2−^, NO_3_^−^, etc.) terminal electron acceptors and/or high ratio of biochemical reduction to electrochemical oxidation rate, an accumulation of the reduced molecules e.g., NADH_2_ in the cytoplasm and/or cytochromes across outer membrane took place. That in turn led to a local concentration of the negative charge, which thermodynamically favored the electron flux from biofilm to the working electrode at this low potential. This has been observed as a favorable property for increasing power output of MFC and labeled self-charging (Caizán-Juanarena et al. 2020). Although in MFC this is heightened, because charging is done under open circuit conditions as opposed to an arbitrary albeit low/negative potential, here.

**Fig. 4:**
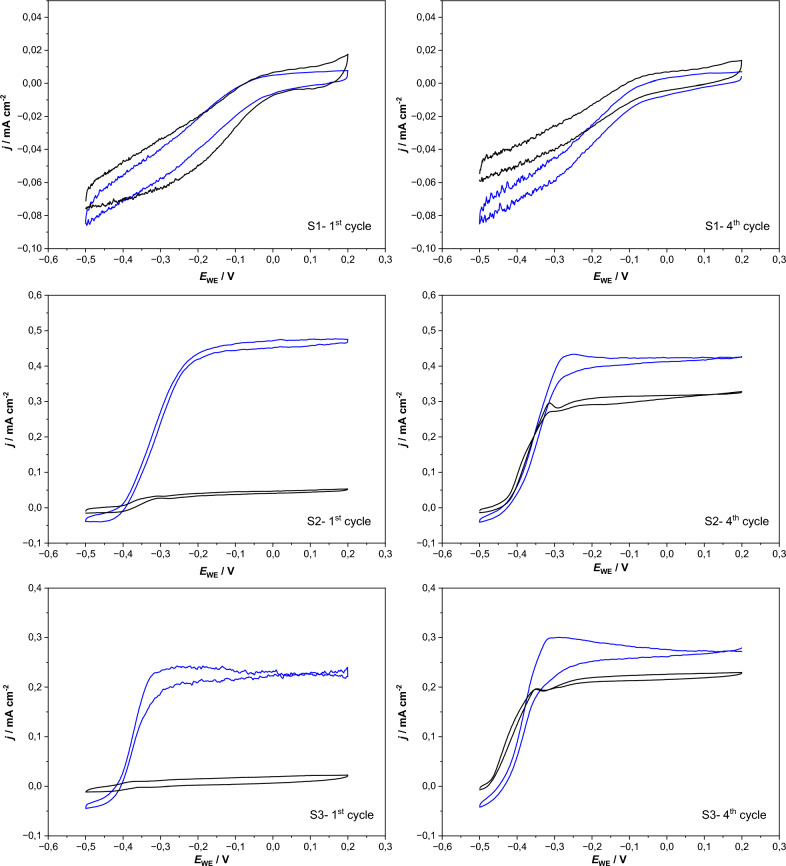
The representative cyclic voltammograms obtained for S1, S2 and S3-BES over the course of HBPR after each polarization stage. left – 1st cycle, right – 4th cycle. Black line – after E_WE_ = − 0.5 V; blue line – after E_WE_ = + 0.2 V

Figure 4 shows single representative cyclic voltammograms obtained after polarization at − 0.5 V or + 0.2 V, respectively, from the 1st and 4th batches of HBPR experiment for S1-, S2- and S3-BES. The averaged values for each triplicate are shown for comparison on Additional file 1: Fig. S2. There is a clear difference in the electrochemical responses of S1-BES compared to both S2 and S3, which are fairly similar. From the 1st to the 4th batch all reactors showed increased capacity to store charge and to immediately go into a reductive charge or oxidative discharge at the respective applied potential. This is particularly evident at the end in the 4th batch. S2 and S3-BES have equilibrium potentials close to − 0.5 V, resulting in sharp increase in current already at very low potentials (− 0.49 V). To a lesser degree this is also true in reverse for the S1-BES, which show increased reduction currents after being polarized at + 0.2 V, approx. 0.1 V more than their equilibrium potential.

From CV conducted with the OC-control WE, examples are shown in Additional file 1: Fig. S3, the formation of hydrogen at the other WE in the range of applied potentials is dismissed as a sink for reductive current. In particular this is clearly observed, i.e., no significant faradaic reductive current in the S3-OC curves.

Based on the analysis of the voltammograms and their respective first derivatives, the formal potentials (${E}^{0{\prime}}$) of the EET components were determined. Most voltammograms had two inflection points, evident through extrema in the 1st derivatives, or peak systems in the case of CVs from non-turnover states.

If not stated otherwise, in the following section the results are being compared against the work of Kracke et al. (Kracke et al. 2015). For a comparison of redox potentials, the reader is reminded that operating conditions in this study differ from standard biochemical conditions (T = 25 °C, P = 1atm, pH = 7). Also note that a number of the proposed redox components were tagged based solely on redox potential, without the confirmation via chemical analysis. Some have only been confirmed to be a part of the microbial inner electron transport chain. However, cellular perforation and/or lysis resulting from continuous polarization switching, could liberate these redox components into the medium. In depth chemical analysis (with e.g., LC–MS-MS) is required to investigate this.

At the beginning of the HBPR experiment (1st batch) a limited number of potential redox pairs involved in EET were tentatively identified for S1, S2 and S3-BES after polarization at + 0.2 and –0.5 V. While voltammograms of S1-BES revealed redox potentials of − 210, − 120 and + 170 mV after applying − 0.5 V and − 210, − 100, and + 140 mV after applying + 0.2 V. Redox potentials characteristic for S2 and S3-BES were more negative. For S2 these redox potentials were − 370 and − 240 mV after − 0.5 V polarization and − 310 and − 20 mV after + 0.2 V polarization, whereas for S3 the redox potentials were − 397, − 303 and + 195 mV and − 375 and, − 300 mV, respectively.

Continuous operation of the BES under the conditions of polarity switching led to further changes in the recorded voltammograms. The analysis of both the respective diagrams and their first derivatives for 4^th^ cycle revealed the appearance of the new reduction and oxidation peaks (inflection points) alongside the existing (identified in batch 1) ones. The potential window (i.e., a specific redox-potential-window in which microbial electron transport chains are able to operate) for all tested systems expanded toward even more negative values. The redox potentials were − 434, − 461 and − 487 mV for S1, S2 and S3, respectively. These findings show that the microorganisms slowly adjusted their electron transfer strategy/capabilities after prolonged exposure to different applied potentials. Similar behavior was previously observed in another study (Yu et al. 2015). This could mean the involvement of redox pairs such as NAD^+^/NADH or due to the very similar standard potentials of Phenazine-1 and FAD^+^/FADH_2_. The former was determined from S3 and the latter from S1 voltammograms. Other observed changes in all systems include the appearance of a redox pair with a potential characteristic of a Riboflavin, indicating an activation of the previously unused FMN^+^/FMNH_2_ system in S1 as well as cytochromes A in S2 & S3. Also, very small peaks with a redox potential of − 270 mV, which is close to standard potential of Menaquinone/Menaquinol pair, was recorded for S3.

By assigning known redox pairs to those redox potentials, it became noticeable, that some redox pairs are active in all systems (here assumed to be cytochromes C and cytochromes B), whereas other are specific either to S1-BES only (cytochromes A, ubiquinone/ubiquinol, rubredoxin) or S2-BES (FMN^+^/FMNH_2_ and Procyanin)) or S3-BES (FMN^+^/FMNH_2_) (Kracke et al. 2015). These findings could be related to either an enrichment of different microorganisms in the respective reactors and/or a biologic response to environmental stress (adaptation to polarization switching). Both A-type cytochromes and ubiquinone/ubiquinol have been shown to be a part of EET in aerobes (e.g., *P. aeruginosa*) and facultative anaerobes (e.g., *S. oneidensis, E. coli*). Oxidized/reduced flavin mononucleotide (FMN^+^/FMNH_2_), which to the best of the authors knowledge, has been identified mainly in anaerobic conditions and was intensively studied as the major endogenous secreted electron shuttles by *Shewanella* sp*.* (Marsili et al. 2008; Von Canstein et al. 2008; Covington et al. 2010; Kotloski and Gralnick 2013; Paquete et al. 2014). Moreover, revealing the presence of Ubiquinone/Ubiquinol in S1-BES might help to explain the observed biofilm detachment from two of the three electrodes and the associated appearance of orange-reddish color of the S1 media and walls on the reactors, see Additional file 1: Fig. S4. This coloration of the media could be from e.g., ubiquinone or flavin. The oxidized form of ubiquinone-10 is orange-colored (Lambrechts and Siebrecht 2013), flavins are yellow-colored when oxidized, red in the semi-reduced anionic state, blue in the neutral (semiquinone) state, and colorless when totally reduced (Silwal and Lu 2018).

Also it should not be assumed that comparable redox potentials for inward and outward EET are evidence for similar (or the same) metabolic pathways/mechanisms, since previous research indicates distinct/individual mechanisms for current production and consumption (Dumas et al. 2008; Strycharz et al. 2008).

### Periodic polarization reversal

To further develop the BES and investigate, if bidirectional EET could be induced within the systems, the experimental procedure was adjusted. S1-BES were first operated and − 0.5 V, then switched to + 0.2 V, whereas S2 and S3 were poised at + 0.2 V first and then switched to − 0.5 V. The switches in polarization occurred after 1/6^th^ of a batch.

**Fig. 5:**
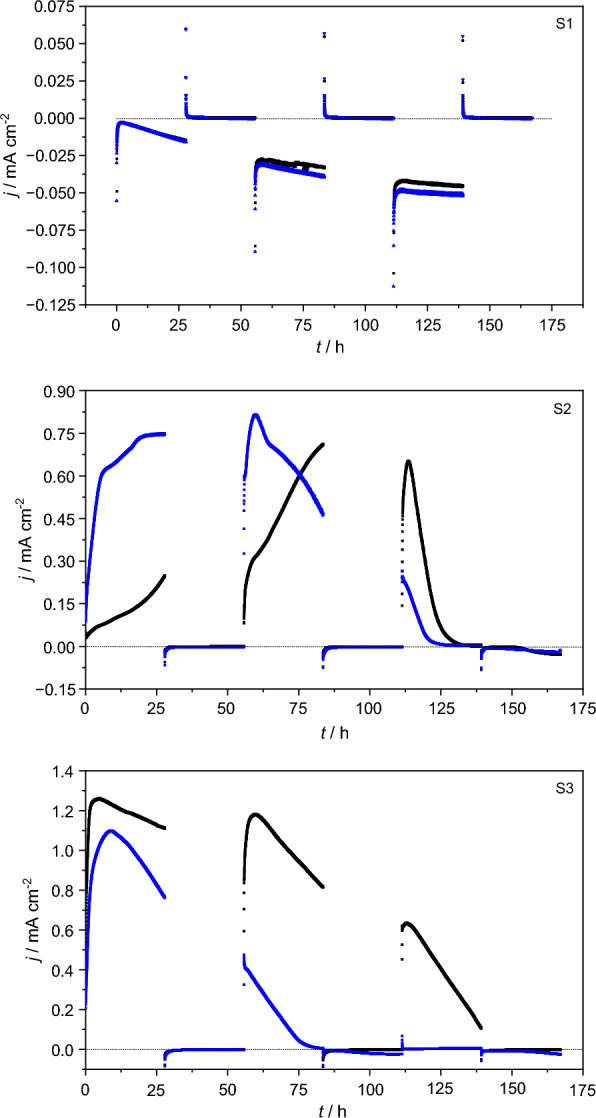
The observed changes in current density in relation to the carbon source concentration obtained for one of the S1, S2 and S3 BES during PPR experiment. Black dots—higher concentration (2^nd^ cycle), blue dots—lower concentration (8^th^ cycle)

Periodic polarization reversal with the concentration of carbon sources as in HBPR did not affect the outcome, i.e., the tested systems demonstrated similar behavior in terms of current generation. As shown in Fig. 5, again only the S1-BES yielded reductive current, whereas only S2 & S3 generated oxidative current.

Again, with the S3 triplicate on average having the highest performance (see Additional file 1: Fig. S5A). As shown in Additional file 1: Fig. S5B, on average the S2 and S3 BES didn’t consume significantly different amounts of charges during polarization at − 0.5 V. The increased oxidative current output of the S3-BES is likely related to the increased buffer capacity (Torres et al. 2008; Yang et al. 2021) in S3 BES with 50 mM phosphate and 30 mM carbonate as opposed to only phosphate in S2-BES.

Reductive current in S1-BES was still likely linked to mediated EET. As stated above, also see Additional file 1: Fig. S4, the orange-reddish coloration of the medium and the surfaces of the S1 H-cells continued to persist and even intensified.

Although no significant bidirectional electron transfer occurred between the electrode and the EAB, a slight but measurable and visible reductive current began to appear after the last polarization reversal during the 2^nd^ batch in the S2-BES (see Fig. 5, black dots). This phenomenon, however, did not repeat itself in the following 3 batches. Surprisingly, also during periodic polarization, the evolution of oxidative current during polarization at -0.5 V occurred, and similarly to HBPR distinctive of S3-BES (Additional file 1: Fig. S6). This time, however, this effect was more evident, with oxidative current densities reaching 0.19 mA cm^−2^. Such a behavior could be assigned to a capability of EAB for storing charge in self-produced redox proteins, and thus exhibiting a pseudo-capacitive behavior, resulting in higher charge storage capacity as well as higher electron mobility across the biofilm. This was already proven to be the outcome of periodic polarization and as such the upregulation of the expression of heme-containing redox proteins associated with the matrix (e.g., c-type cytochromes) (Zhang et al. 2018).

In HBPR and in the first 5 batches of PPR the acetate fed BES did not reach a non-turnover state after oxidative current generation during polarization at + 0.2 V. This is likely due to the nominal oxidation phase i.e., polarization at + 0.2 V being too short or alternatively the concentration of acetate was “too high” for the relative amount of EAB biomass on the electrodes, compared to previous studies inoculated from the same BES-mother reactor and fed with 10 mM acetate (Alvarez Esquivel et al. 2020). Apart from this, HPLC analysis showed trace amounts of ethanol, propionate, butyrate as well as succinate and some unidentified substances, see SI-HPLC results i.e., Additional file 1: Table S1and S2 for more details. These results indicate that when CO_2_/carbonate was reduced, it was primarily assimilated, whereby the identified und unidentified substances were likely mediators excreted into the media or were intermediates or decay products. In some cases, in PPR acetate was found at the end of a batch although the respective BES had reached a non-turnover state during polarization at + 0.2 V prior to polarization at − 0.5 V, which could also indicate that CO_2_/carbonate was reduced to acetate.

Based on the observed results, it was decided that the availability of acetate was preventing the S2 and S3-BES from switching to an electroautotrophic metabolism. C-source availability as a limiting factor is well known, e.g., dissolved CO_2_ concentration limits the growth of chain elongating microorganisms (Tomlinson and Barker 1954). In these experiments it is not a question of availability, but a question of metabolic priority. C-source and energy source priority is discussed in terms of organism specific diauxie vs. co-utilization and priority is related to which has the higher biomass yield and faster growth rates (Perrin et al. 2020; Salvy and Hatzimanikatis 2021).

**Fig. 6:**
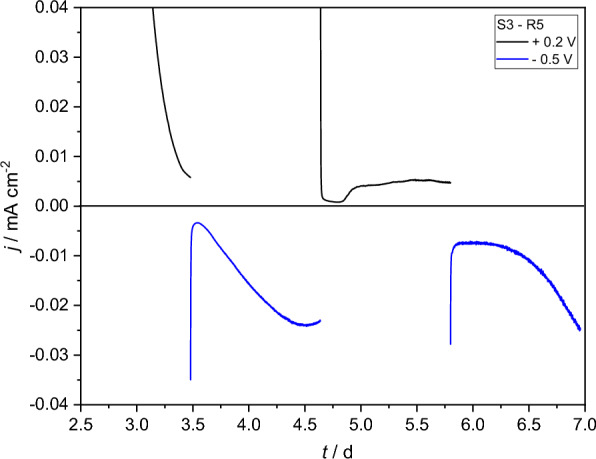
Zoomed in section of the 8^th^ batch from Fig. 5 of R5 in the S3 triplicate showing reversible bidirectional electron transfer

Therefore, the concentration of all carbon sources was reduced by 50% to shorten the time in which acetate in particular was available. Decreasing the carbon source concentrations proved partially successful. As shown in Fig. 5 S1-BES did not develop any oxidative current, S2 and S3-BES showed reductive current at - 0.5 V when acetate was depleted, indicated by a non-turnover state being reached during prior polarization at + 0.2 V. However, true bidirectional EET was only observed in one BES, which is shown in Fig. 6.

The reductive performance of S1-BES fed only with carbonate improved, i.e., more negative current densities were achieved by the end of each batch. Interestingly, the system never reached its maximum electron consumption, i.e., no negative peak was observed. After each batch the medium was replaced and reductive current started at 0 and slowly increased, which is further evidence of the use of mediated EET by biomass on the glass walls/in the media of the reactors. Therefore, also the most logical explanation for the lack of mediated oxidative electrode respiration in S1 seems to be a presence of alternate electron acceptor(s) the medium. Generation of reductive current during electrode polarization at − 0.5 V resulted in oxygen production at counter electrode followed by its possible diffusion toward working electrode compartment. This dissolved oxygen and the sulfate present in the media could serve as alternative electron acceptors for an/aerobic microorganisms suspended in media or attached to the glass wall of the chamber.

In contrast to other studies published so far, in which bidirectional (Yates et al. 2017; Izadi et al. 2021) as well as switching (Pous et al. 2016) electron transfer was demonstrated, polarization switching applied to the heterotrophically cultivated mixed EAB biofilms in this study fed solely on inorganic carbon source (carbonate) did not elicit bidirectional EET, but a switch from oxidative, during cultivation, to reductive activity during HBPR occurred. Bidirectional EET was only observed in one mixed culture EAB biofilm fed with mixed carbon sources. Reasons for these developments could be the choice of inoculum compared to the obtained heterotrophically cultivated mixed marine sediment EAB (Izadi et al. 2021) or the length of exposure to polarization switching (Yates et al. 2017). Also various substrates can lead to formation of different metabolic networks, biochemical conversions and electron transfer means (Raes et al. 2020). Syntrophic metabolism (interspecies (IET), direct (DET) or mediated (MET) electron transfer) could also contribute to the lack of reductive current (Kouzuma et al. 2015). Syntrophic acetate catabolism could occur, when both acetate oxidizing and CO_2_ reducing species were present in the BES, as proven for *Geobacter* and *Methanosarcina* species (Kato et al. 2012).

### Microbial community analysis

**Fig. 7:**
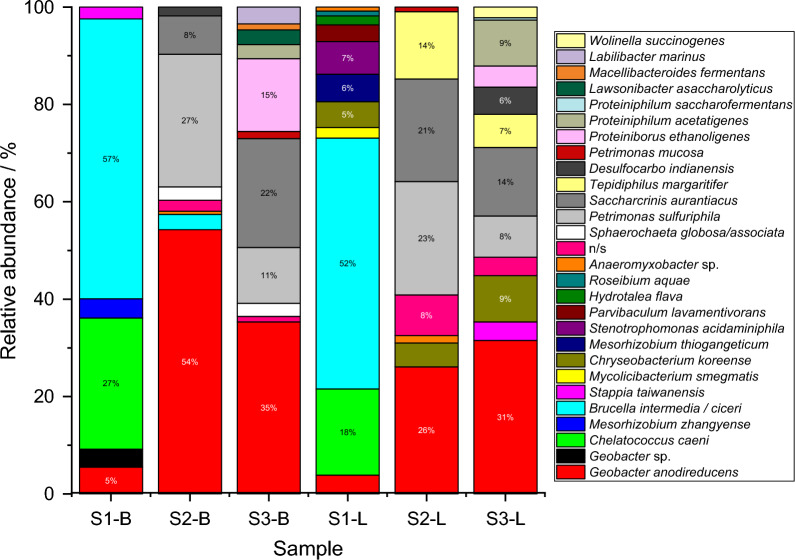
The relative abundance of the microbial communities in biofilm samples (B) and depleted media/BES wall (L) in relation to the carbon source (S1, S2 & S3) after completion the last experiment. n/s—no similarities

The microbial communities in the developed biofilms and in the depleted media and/or from walls of the BES were analyzed to identity of the enriched microorganisms vs. the composition of the used inoculum and relation to the experimental conditions. The DNA gel images obtained as a result of DNA profile generation are presented in the Additional file 1: Fig. S11. Only one S1-BES had biofilm on the electrode (see Additional file 1: Fig. S12). Moreover, the biofilm was rather thin and not evenly spread. In contrast, biofilms from all S2 and S3 electrodes (Additional file 1: Figs. S13, S14, respectively) could be characterized as thick and covering most of the electrodes. The relative abundance of all detected and identified species in the inoculum and phosphate buffer used for media preparation are shown on Additional file 1: Fig. S15, whereas all analyzed samples from the BES are shown in Fig. 7.

As expected, the results show that biofilm used as the inoculum source for all BES was dominated by one species (39.5%), namely *Geobacter anodireducens*. This microorganism is one of the most widely studied electroactive bacteria capable of formation thick and stable biofilms. Other anaerobic bacteria represented by e.g., *Rectinema cohabitans* and *Seramator thermalis* were relatively less abundant (21% and 17%, respectively) and so far were not proved to be electroactive. Slightly more differentiated was phosphate buffer solution (PBS) microbiome, in which *H. flava*, *B. elkanii, H. penzbergensis*, *C. necator*, *M. erdmani* and an unidentified *Sinobacteraceae* were identified in comparable numbers: i.e., 16% for two first, 14% for next two and 11% for the last two mentioned species. Surprisingly, only *G. anodireducens* and *H. flava* were found afterwards in BES biofilm and/or liquid samples.

A large number of other microorganisms were successfully enriched in S1, S2 and S3-BES, which were previously undetected in the inoculum or in the PBS, see Additional file 1: Fig. S15. This is evidence that the chosen method of cultivation/operation exerts a high selection pressure, allowing species to flourish that were previously below the limit of detection. The obtained results show two major differences between the tested samples: Firstly, clear evidence was found for the domination of *Geobacter anodireducens* for S2 and S3 samples. Secondly, S1-BES were dominated by *Brucella intermedia* and/or *ciceri*, which constituted over 50% of population, followed by *Chelatococcus caeni* with about 20% and *Geobacter* sp. only a maximum 5%. Among S2 & S3 microbiome, *Petrimonas sulfuriphila* and *Saccharicrinis aurantiacus* were identified in larger quantities in both biofilm and liquid media, however, the first one was more abundant for S2 and the latter one for S3 samples as well as *Proteiniborus ethanoligenes* in the S3 biofilms.

While *Geobacter* sp. are well-known electroactive microorganisms capable of bidirectional electron transfer, to the best of authors knowledge the *Brucella* sp. found here has not been detected/used explicitly in microbial electrochemical technologies (MET). *Chelatococcus caeni* utilizes the mediator ubiquinone-10 in its electron transport chain for aerobic respiration, which would be consistent with the other results in this study mentioned previously. Interestingly, both species are capable of nitrate reduction, which suggest their capability to adapt to switch to anaerobic respiration.

*P. sulfuriphila* is strictly anaerobic chemoorganotroph, capable of utilizing acetate as a possible carbon source and elemental sulfur or nitrate as electron acceptors (Grabowski et al. 2005). *S. aurantiacus* is facultatively anaerobic bacterium capable of fermentative metabolism. For both species menaquinones were identified as the major respiratory quinones used to conduct respiration, which explains the identification of Menaquinone/Menaquinol redox pair in S3 voltammograms during HBPR experiment. To date their application for MET has not been studied.

*Proteiniborus ethanoligenes and Proteiniphilum acetatigenes* as well as *Tepidiphilus margaritifer* and *Chryseobacterium koreense* were relatively abundant in S2 & S3 (> 7%). None of them, however, was detected in S1. The first two bacterial species can be characterized as mesophilic anaerobes previously found in granular sludge used for treatment of either food industry or brewery wastewater, respectively. *P. ethanoligenes* produces ethanol whereas the *P. acetatigenes* is known to produce acetate (Chen and Dong 2005). *P. acetatigenes*, however, was shown not to be exoelectrogenic (Logan et al. 2019). On the other hand, *T. margaritifer* and *C. koreense* are aerobic bacteria that can be useful for MET applications (Yang et al. 2020). The former was already proved to be a dominant species in acetate-based MFC (Dessì et al. 2019), however, in our study *T. margaritifer* was found only in planktonic form suspended in the BES media. *C. koreense* together with *Stenotrophomonas nitritireducens* were previously found to be the dominant species of microbial consortia at Sb(V)-reducing biocathodes (Nguyen et al. 2019).

Several other microorganisms were detected in the samples, but they were present only in small numbers. According to the available literature at the time of submission, at least a few of those species are capable of extracellular electron transfer and direct interspecies electron transfer. *Sphaerochaeta globosa* and *S. associata* found in biofilm samples of S2 & S3 are strictly fermentative and anaerobic bacteria, producing ethanol, acetate and formate as major fermentation end products (Ritalahti et al. 2011). These bacteria are also capable of reducing Fe(III) oxides through extracellular electron transfer (Li et al. 2020). Strictly anaerobic, sulfate-reducing bacterium *Desulfocarbo indianensis* was identified in S2-B and S3-L samples. According to An and Picardal (2014) this microorganism can grow either autotrophically with hydrogen as the electron donor or heterotrophically on organics such as acetate, formate, pyruvate, butyrate, fumarate, succinate, benzoate, and palmitate (An and Picardal 2014), but without allowing for fermentative growth. *Lawsonibacter asaccharolyticus* identified in S3 biofilm is an obligate anaerobe that can produce acetate and butyrate as metabolic end-products (Sakamoto et al. 2018).

The detection of the strain *Wolinella succinogenes* in S3 media can be very promising concerning syntrophic cocultures. This microorganism is capable of syntrophic acetate degradation when cocultured with *G. sulfurreducens* with nitrate as the electron acceptor (Cord-Ruwisch et al. 1998). In this case a separate carrier system other than hydrogen can be employed for electron transfer (Kaden et al. 2002).

Characteristic only for S2 samples and S1 liquid media was iron-reducing bacterium *Anaeromyxobacter* sp. This anaerobe utilizes acetate, lactate, and pyruvate as electron donors, and nitrate and iron (III) as electron acceptors. The bacterium grows very slowly with e.g., ethanol and appears to be oligotrophic.

Among the species that were found only in the S1-L samples, the relative abundance of only two of them, namely *Stenotrophomonas acidaminiphila* and *Mesorhizobium thiogangeticum*, exceeded 5%. Both microorganisms are aerobic, gram-negative proteobacteria. The latter one is capable of oxidizing thiosulfate and elemental sulfur for chemolithoautotrophic growth as well as of utilizing succinate as carbon source (Alam et al. 2021). The former has the ability to utilize PAHs (pyrene and phenanthrene) as an energy and carbon source (Mangwani et al. 2014). The abundance of other identified species, e.g., *Mycolicibacterium smegmatis*, *Hydrotalea flava* and *Parvibaculum lavamentivorans* was lower than 5% and none of them are known to be electroactive. *Mesorhizobium zhangyense* was identified only in S1-B sample. It is an aerobic bacteria capable of utilizing, e.g., pyruvic acid, acetate as well as succinate as sole carbon sources as well as assimilating citric acid (Xu et al. 2018). Similar to *Chelatococcus caeni* this bacterium also utilizes the mediator ubiquinone-10 in its electron transport chain for aerobic respiration, which is in line with redox potentials determined with CV.

Another important feature of the examined samples is the significant domination of aerobic over anaerobic microorganisms in the S1-BES. Aerobes constituted 84.8% of S1 biofilm and 94.3% of the S1-L populations, whereas completely the opposite has been determined for S2 and S3 communities. In the BES fed with organic (S2) and mixed (S3) carbon source mostly anaerobic bacteria were enriched, with their slightly elevated abundance detected in biofilms in comparison to the liquid media. For S2 the maximum share of anaerobic microorganisms in population was 96.9% for biofilms and 81.7% for L-samples, whereas for S3 those values were 96.5% and 80%, respectively. The enrichment of aerobic species in S1 samples can be explained by the oxygen availability and, therefore, unfavorable conditions for anaerobic growth. Oxygen was generated through water electrolysis at the counter electrode as the compensation for reductive current observed at the WE. The penetration of oxygen towards WE chamber was to certain degree limited by the CEM separating both chambers; nevertheless, the flow was not completely avoided and eventually served as a terminal electron acceptor. In the case of S2 & S3-BES the system was able to maintain anoxic conditions due to the lack of reductive current at the WE.

These findings can shed more light into the metabolic pathway utilized by bacteria enriched in tested systems. In anaerobic environments (i.e., S2 & S3) the reverse ß-oxidation, the reverse Krebs cycle and the Wood-Ljungdahl pathways should be dominant for biosynthesis or carbon fixation, carbon fixation under aerobic conditions (S1) can be operated only through the 3-hydroxypropionate bicycle or 3-hydroxypropionate-4-hydroxybutyrate cycle with ubiquinone as electron acceptor (Bar-Even et al. 2012). This in turn is again partially reflected in the S1 cyclic voltammetry results and allows to speculate that yet unidentified compound found in S1 HPLC analysis may actually be an intermediate or product of the 3-hydroxypropionate bicycle.

## Conclusions

The study investigated if mixed EAB cultures cultivated at continuously at + 0.2 V with acetate could be adapted from oxidative to reductive or bidirectional EET. To this end, after cultivation, a periodic potential reversal regime between − 0.5 and + 0.2 V was applied in combination with a culture medium containing either carbonate, acetate or a mixture of the two as carbon sources. The three triplicates had EAB mixed cultures which differentiated over the course of the experiments in equal measure as their electrochemical behavior. Substrate or c-source priority i.e., diauxie limits mixed EAB cultures from adapting or transitioning to bidirectional EET based metabolism, but it is possible for them to transition to at least electroautotrophic activity, whereby further detailed investigation, e.g., using LC–MS/MS for identification of mediators, should be carried out.

This study paves the way for more sophisticated investigation. Future investigations could: (i) Answer the questions of whether or not mediated EET can be bidirectional; (ii) Investigate the triggers for EAB biofilm detachment; (iii) Investigate bidirectional EET in BES with or without oxygen evolution at the counter electrode (iv) Further develop and/or optimize cultivation procedures for bidirectional EAB.

### Supplementary Information

**Additional file 1.** Additional tables and figures.

## Data Availability

There is no additional data available, beyond the results shown in this manuscript and the supporting information, in Additional file 1, to it.

## References

[CR1] Alam M, Fernandes S, Mandal S (2021). 34S enrichment as a signature of thiosulfate oxidation in the “Proteobacteria”. FEMS Microbiol Lett.

[CR2] Alvarez Esquivel DY, Brown RK, Knohl S, Schröder U (2020). Developing cheap and mass-producible graphite-filled paper as an anode material for microbial electrochemical technologies. ChemElectroChem.

[CR3] An TT, Picardal FW (2014). *Desulfocarbo indianensis* gen. nov., sp. nov., a benzoate-oxidizing, sulfate-reducing bacterium isolated from water extracted from a coal bed. Int J Syst Evol Microbiol.

[CR4] Balch WE, Fox GE, Magrum LJ (1979). Methanogens: reevaluation of a unique biological group. Microbiol Rev.

[CR5] Bar-Even A, Noor E, Milo R (2012). A survey of carbon fixation pathways through a quantitative lens. J Exp Bot.

[CR6] Baudler A, Riedl S, Schröder U (2014). Long-term performance of primary and secondary electroactive biofilms using layered corrugated carbon electrodes. Front Energy Res.

[CR7] Cabau-Peinado O, Straathof AJJ, Jourdin L (2021). A general model for biofilm-driven microbial electrosynthesis of carboxylates from CO2. Front Microbiol.

[CR8] Caizán-Juanarena L, Borsje C, Sleutels T (2020). Combination of bioelectrochemical systems and electrochemical capacitors: Principles, analysis and opportunities. Biotechnol Adv.

[CR9] Chatterjee P, Dessì P, Kokko M (2019). Selective enrichment of biocatalysts for bioelectrochemical systems: a critical review. Renew Sustain Energy Rev.

[CR10] Chen S, Dong X (2005). *Proteiniphilum acetatigenes* gen. nov., sp. nov., from a UASB reactor treating brewery wastewater. Int J Syst Evol Microbiol.

[CR11] Chong GW, Karbelkar AA, El-Naggar MY (2018). Nature’s conductors: what can microbial multi-heme cytochromes teach us about electron transport and biological energy conversion?. Curr Opin Chem Biol.

[CR12] Cord-Ruwisch R, Lovley DR, Schink B (1998). Growth of *Geobacter sulfurreducens* with acetate in syntrophic cooperation with hydrogen-oxidizing anaerobic partners. Appl Environ Microbiol.

[CR13] Covington ED, Gelbmann CB, Kotloski NJ, Gralnick JA (2010). An essential role for UshA in processing of extracellular flavin electron shuttles by *Shewanella oneidensis*. Mol Microbiol.

[CR14] Dessì P, Chatterjee P, Mills S (2019). Power production and microbial community composition in thermophilic acetate-fed up-flow and flow-through microbial fuel cells. Bioresour Technol.

[CR15] Dumas C, Basseguy R, Bergel A (2008). Microbial electrocatalysis with *Geobacter sulfurreducens* biofilm on stainless steel cathodes. Electrochim Acta.

[CR16] Fricke K, Harnisch F, Schröder U (2008). On the use of cyclic voltammetry for the study of anodic electron transfer in microbial fuel cells. Energy Environ Sci.

[CR17] Grabowski A, Tindall BJ, Bardin V (2005). *Petrimonas sulfuriphila* gen. nov., sp. nov., a mesophilic fermentative bacterium isolated from a biodegraded oil reservoir. Int J Syst Evol Microbiol.

[CR18] Hartline RM, Call DF (2016). Substrate and electrode potential affect electrotrophic activity of inverted bioanodes. Bioelectrochemistry.

[CR19] Ikeda S, Takamatsu Y, Tsuchiya M (2021). *Shewanella oneidensis* MR-1 as a bacterial platform for electro-biotechnology. Essays Biochem.

[CR20] Izadi P, Gey MN, Schlüter N, Schröder U (2021). Bidirectional electroactive microbial biofilms and the role of biogenic sulfur in charge storage and release. iScience.

[CR21] Jiang Y, Zeng RJ (2019). Bidirectional extracellular electron transfers of electrode-biofilm: Mechanism and application. Bioresour Technol.

[CR22] Jourdin L, Sousa J, van Stralen N, Strik DPBTB (2020). Techno-economic assessment of microbial electrosynthesis from CO2 and/or organics: An interdisciplinary roadmap towards future research and application. Appl Energy.

[CR23] Kaden J, Galushko SA, Schink B (2002). Cysteine-mediated electron transfer in syntrophic acetate oxidation by cocultures of Geobacter sulfurreducens and Wolinella succinogenes. Arch Microbiol.

[CR24] Karthikeyan R, Singh R, Bose A (2019). Microbial electron uptake in microbial electrosynthesis: a mini-review. J Ind Microbiol Biotechnol.

[CR25] Kato S, Hashimoto K, Watanabe K (2012). Methanogenesis facilitated by electric syntrophy via (semi)conductive iron-oxide minerals. Environ Microbiol.

[CR26] Kotloski NJ, Gralnick JA (2013). Flavin electron shuttles dominate extracellular electron transfer by Shewanella oneidensis. Mbio.

[CR27] Kouzuma A, Kato S, Watanabe K (2015). Microbial interspecies interactions: Recent findings in syntrophic consortia. Front Microbiol.

[CR28] Kracke F, Vassilev I, Krömer JO (2015). Microbial electron transport and energy conservation—the foundation for optimizing bioelectrochemical systems. Front Microbiol.

[CR29] Kumar A, Hsu LHH, Kavanagh P (2017). The ins and outs of microorganism-electrode electron transfer reactions. Nat Rev Chem.

[CR30] Lambrechts P, Siebrecht S (2013). Coenzyme Q10 and ubiquinol as adjunctive therapy for heart failure. Agro Food Ind Hi Tech.

[CR31] Li Y, Liu M, Che X (2020). Biochar stimulates growth of novel species capable of direct interspecies electron transfer in anaerobic digestion via ethanol-type fermentation. Environ Res.

[CR32] Li Z, Chang W, Cui T (2021). Adaptive bidirectional extracellular electron transfer during accelerated microbiologically influenced corrosion of stainless steel. Commun Mater.

[CR33] Liang D, Li Z, Liu G (2023). Construction of bidirectional electron transfer biofilms via periodic polarity reversal. Chem Eng J.

[CR34] Logan BE, Rossi R, Ragab A, Saikaly PE (2019). Electroactive microorganisms in bioelectrochemical systems. Nat Rev Microbiol.

[CR35] Mangwani N, Shukla SK, Kumari S (2014). Characterization of *Stenotrophomonas acidaminiphila* NCW-702 biofilm for implication in the degradation of polycyclic aromatic hydrocarbons. J Appl Microbiol.

[CR36] Marsili E, Baron DB, Shikhare ID (2008). Shewanella secretes flavins that mediate extracellular electron transfer. Proc Natl Acad Sci U S A.

[CR37] Mickol RL, Eddie BJ, Malanoski AP (2021). Metagenomic and metatranscriptomic characterization of a microbial community that catalyzes both energy-generating and energy-storing electrode reactions. Appl Environ Microbiol.

[CR38] Nguyen VK, Park Y, Lee T (2019). Microbial antimonate reduction with a solid-state electrode as the sole electron donor: a novel approach for antimony bioremediation. J Hazard Mater.

[CR39] Okamoto A, Hashimoto K, Nealson KH (2014). Flavin redox bifurcation as a mechanism for controlling the direction of electron flow during extracellular electron transfer. Angew Chemie - Int Ed.

[CR40] Paquete CM, Fonseca BM, Cruz DR (2014). Exploring the molecular mechanisms of electron shuttling across the microbe/metal space. Front Microbiol.

[CR41] Paquete CM, Morgado L, Salgueiro CA, Louro RO (2022). Molecular mechanisms of microbial extracellular electron transfer: the importance of multiheme cytochromes. Front Biosci Landmark.

[CR42] Pereira J, Mediayati Y, van Veelen HPJ (2022). The effect of intermittent anode potential regimes on the morphology and extracellular matrix composition of electro-active bacteria. Biofilm.

[CR43] Perrin E, Ghini V, Giovannini M (2020). Diauxie and co-utilization of carbon sources can coexist during bacterial growth in nutritionally complex environments. Nat Commun.

[CR44] Pous N, Carmona-Martínez AA, Vilajeliu-Pons A (2016). Bidirectional microbial electron transfer: switching an acetate oxidizing biofilm to nitrate reducing conditions. Biosens Bioelectron.

[CR45] Rabaey K, Rozendal RA (2010). Microbial electrosynthesis—revisiting the electrical route for microbial production. Nat Rev Microbiol.

[CR46] Raes SMT, Jourdin L, Buisman CJN, Strik DPBTB (2020). Bioelectrochemical chain elongation of short-chain fatty acids creates steering opportunities for selective formation of n-Butyrate, n-Valerate or n-Caproate. ChemistrySelect.

[CR47] Riedl S, Brown RK, Alvarez Esquivel DY (2019). Cultivating electrochemically active biofilms at continuously changing electrode potentials. ChemElectroChem.

[CR48] Ritalahti KM, Justicia-Leon SD, Cusick KD (2011). *Sphaerochaeta globosa* gen. nov., sp. nov. and *Sphaerochaeta pleomorpha* sp. nov., free-living, spherical spirochaetes. Int J Syst Evol Microbiol.

[CR49] Rosenbaum M, Aulenta F, Villano M, Angenent LT (2011). Cathodes as electron donors for microbial metabolism: which extracellular electron transfer mechanisms are involved?. Bioresour Technol.

[CR50] Ross DE, Flynn JM, Baron DB (2011). Towards electrosynthesis in Shewanella: energetics of reversing the Mtr pathway for reductive metabolism. PLoS ONE.

[CR51] Saheb-Alam S, Singh A, Hermansson M (2018). Effect of start-up strategies and electrode materials on carbon dioxide reduction on biocathodes. Appl Environ Microbiol.

[CR52] Sakamoto M, Iino T, Yuki M, Ohkuma M (2018). *Lawsonibacter asaccharolyticus* gen. nov., sp. nov., a butyrateproducing bacterium isolated from human faeces. Int J Syst Evol Microbiol.

[CR53] Salvy P, Hatzimanikatis V (2021). Emergence of diauxie as an optimal growth strategy under resource allocation constraints in cellular metabolism. Proc Natl Acad Sci U S A.

[CR54] Silwal AP, Lu HP (2018). Raman spectroscopy probing of redox states and mechanism of flavin coenzyme. J Raman Spectrosc.

[CR55] Soussan L, Riess J, Erable B (2013). Electrochemical reduction of CO2 catalysed by *Geobacter sulfurreducens* grown on polarized stainless steel cathodes. Electrochem Commun.

[CR56] Strycharz SM, Woodard TL, Johnson JP (2008). Graphite electrode as a sole electron donor for reductive dechlorination of tetrachlorethene *by Geobacter lovleyi*. Appl Environ Microbiol.

[CR57] Tomlinson N, Barker HA (1954). Carbon dioxide and acetate utilization by *Clostridium kluyveri*. J Biol Chem.

[CR58] Torres CI, Marcus AK, Rittmann BE (2008). Proton transport inside the biofilm limits electrical current generation by anode-respiring bacteria. Biotechnol Bioeng.

[CR59] Vassilev I, Hernandez PA, Batlle-Vilanova P (2018). Microbial electrosynthesis of isobutyric, butyric, caproic acids, and corresponding alcohols from carbon dioxide. ACS Sustain Chem Eng.

[CR60] Von Canstein H, Ogawa J, Shimizu S, Lloyd JR (2008). Secretion of flavins by Shewanella species and their role in extracellular electron transfer. Appl Environ Microbiol.

[CR61] Xie Q, Lu Y, Tang L (2021). The mechanism and application of bidirectional extracellular electron transport in the field of energy and environment. Crit Rev Environ Sci Technol.

[CR62] Xu L, Zhang Y, Mohamad OA (2018). *Mesorhizobium zhangyense* sp. nov., isolated from wild Thermopsis lanceolate in northwestern China. Arch Microbiol.

[CR63] Yang Y, Ren H, Ben-Tzvi P (2017). Optimal interval of periodic polarity reversal under automated control for maximizing hydrogen production in microbial electrolysis cells. Int J Hydrogen Energy.

[CR64] Yang K, Ji M, Liang B (2020). Bioelectrochemical degradation of monoaromatic compounds: current advances and challenges. J Hazard Mater.

[CR65] Yang G, Mai Q, Zhuang Z, Zhuang L (2021). Buffer capacity regulates the stratification of anode-respiring biofilm during brewery wastewater treatment. Environ Res.

[CR66] Yates MD, Ma L, Sack J (2017). Microbial electrochemical energy storage and recovery in a combined electrotrophic and electrogenic biofilm. Environ Sci Technol Lett.

[CR67] Yong YC, Yu YY, Zhang X, Song H (2014). Highly active bidirectional electron transfer by a self-assembled electroactive reduced-graphene-oxide-hybridized biofilm. Angew Chemie Int Ed.

[CR68] Yu Y, Wu Y, Cao B (2015). Adjustable bidirectional extracellular electron transfer between *Comamonas testosteroni* biofilms and electrode via distinct electron mediators. Electrochem Commun.

[CR69] Yu L, Yuan Y, Rensing C, Zhou S (2018). Combined spectroelectrochemical and proteomic characterizations of bidirectional *Alcaligenes faecalis* electrode electron transfer. Biosens Bioelectron.

[CR70] Zhang X, Prévoteau A, Louro RO (2018). Periodic polarization of electroactive biofilms increases current density and charge carriers concentration while modifying biofilm structure. Biosens Bioelectron.

[CR71] Zou L, Wu X, Huang Y (2019). Promoting shewanella bidirectional extracellular electron transfer for bioelectrocatalysis by electropolymerized riboflavin interface on carbon electrode. Front Microbiol.

