# Supplementary material for: Carbon source priority and availability limit bidirectional electron transfer in freshwater mixed culture electrochemically active bacterial biofilms
Source: Bioresour Bioprocess. 2023 Sep 20;10(1):64. doi: 10.1186/s40643-023-00685-w (PMC10991894; doi:10.1186/s40643-023-00685-w)
Supplement: Supplementary file 1 — Additional file 1. Additional tables and figures. [file 40643_2023_685_MOESM1_ESM.docx]

**Additional file 1**

**To**

Carbon source priority and availability limit bidirectional electron transfer in freshwater mixed culture electrochemically active bacterial biofilms

Karina Anna Michalska, Robert Keith Brown, Uwe Schröder

Institute of Biochemistry, University of Greifswald

Figure S1: The example of resulting current densities obtained for one of the S3-BES during 4^th^ batch of HBPR.

Figure S2: The averaged cyclic voltammograms obtained for S1, S2 and S3-BES over the course of HBPR after each polarization stage (left – after E_WE_ = -0.5 V, right – after E_WE_ = +0.2 V). Black – cycle 1; red – cycle 2; blue – cycle 3, purple – cycle 4.

Figure S3: Exemplary CV obtained at the OC-control WE after on half batch in batch 4 of the HBPR experimental phase.


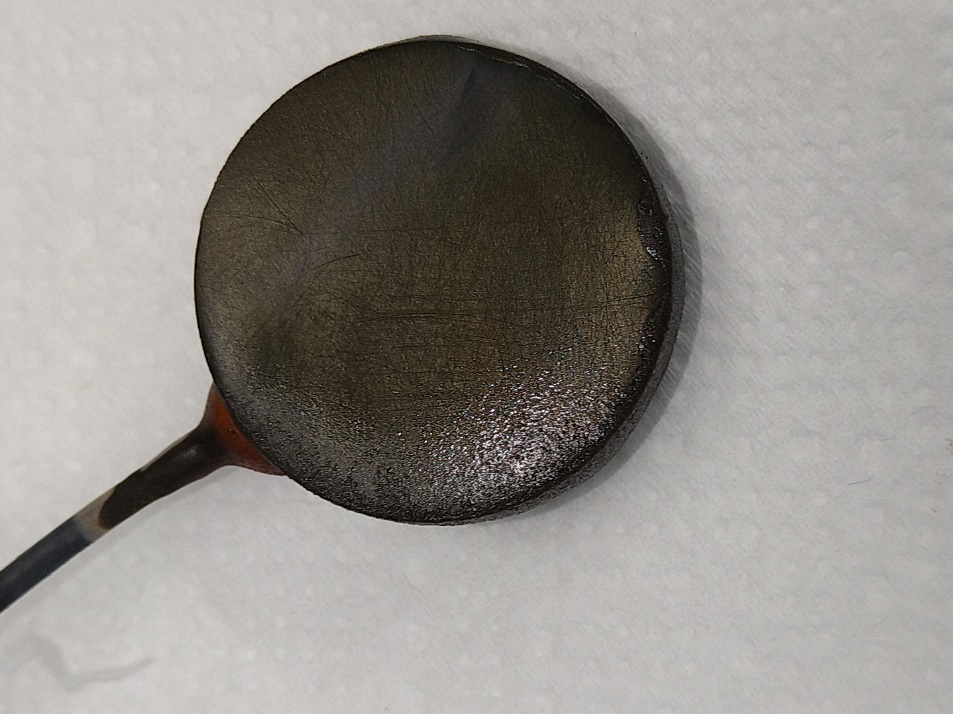

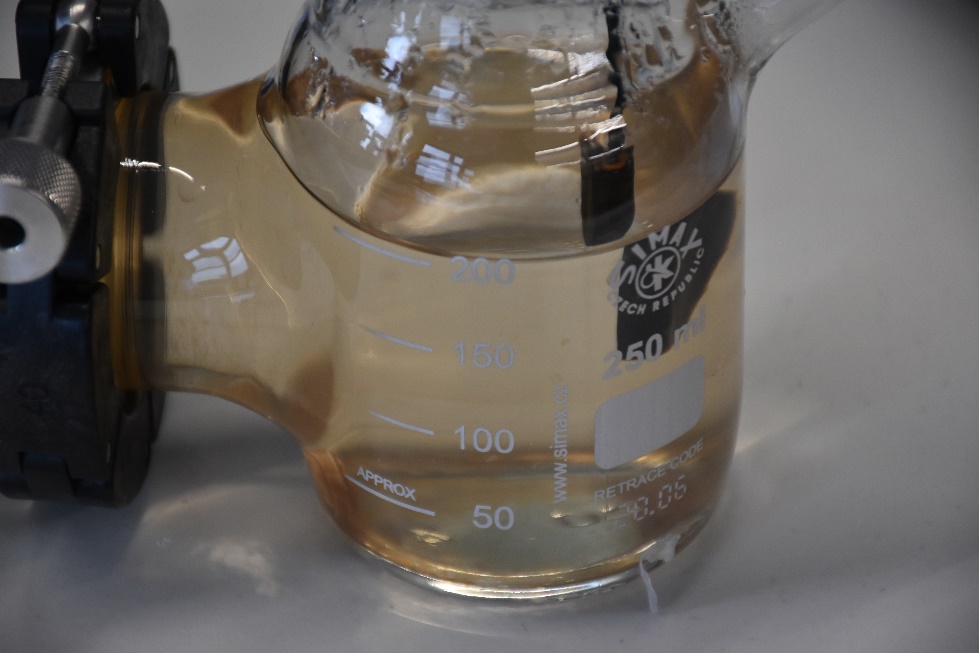


Figure S4: Electrode without noticable biofilm taken from and a photo showing the colouration of media in one of the S1-BES.

Figure S5: A) Average maximum current densities (j_max_) of S2 and S3 BES over the course of all experiments. B) Cumulative charge (Q) over the CA operations at the two applied potentials for S2 & S3 BES.

Figure S6: Oxidative current evolution in a S3-BES during PPR for polarization specifically at -0.5V (black line) as well as, as expected, at +0.2V (blue line).

**SI - HPLC -Results**

In S1-BES three substances were identified, there are three clear peaks; one at a retention time of 9.4 min, one at a retention time of 19.3 min and one at a retention time of 43 min, as shown in Figure S-6 and Figure S-7. The third and second peaks are from components of the vitamin solution and shows up in fresh media samples. The first peak was confirmed in later testing (not shown) to not be a TCA-cycle compound and was only found in S1-BES, which makes it likely that this is the EET mediator indirectly identified by CV and observation coloration changes (section 3.2 and 3.3, main manuscript).

Trace amounts of other more complex/larger compounds were also detected in various samples of all BES at the end of PPR as well as in between and at the end of HBPR batches. The exact identity was not confirmed, however based on the retention times (e.g. see Figure S-9) and being detected with DAD indicates that they are all likely C6+ compounds with at least on carboxylic group.

Table S1: The composition of the supernatants obtained over the course of HBPR experiment after each polarization switch.

| **BES** | | **S1** | | **S2** | | **S3** | |
| --- | --- | --- | --- | --- | --- | --- | --- |
| **Polarization** | | -0.5 V | +0.2 V | -0.5 V | +0.2 V | -0.5 V | +0.2 V |
| **Compo- und** | **Cycle** | mM | mM | mM | mM | mM | mM |
| **Acetic acid** | 1 | 0.0 | 0.0 | 6.205 ± 1.719 | 0.576 ± 0.613 | 5.668 ± 1.245 | 0.303 ± 0.332 |
|  | 2 | 0.0 | 0.0 | 5.792 ± 2.663 | 0.216 ± 0.253 | 6.235 ± 0.931 | 0.065 ± 0.113 |
|  | 4 | 0.0 | 0.0 | 5.695 ± 2.797 | 0.440 ± 0.665 | 6.863 ± 1.024 | 1.757 ± 2.537 |
| **Ethanol** | 1 | 1.055 ± 0.159 | 3.333 ± 0.517 | 1.096 ± 0.548 | 0.694 ± 0.315 | 1.147 ± 0.143 | 3.856 ± 2.613 |
|  | 2 | 0.261 ± 0.205 | 0.419 ± 0.659 | 0.343 ± 0.068 | 0.308 ± 0.123 | 1.174 ± 1.120 | 0.330 ± 0.399 |
|  | 4 | 0.0 | 0.415 ± 0.146 | 2.661 ± 0.402 | 1.612 ± 1.280 | 0.073 ± 0.074 | 0.972 ± 0.278 |
| **Lactic acid** | 1 | 0.0 | 0.0 | 0.0 | 0.0 | 0.0 | 0.0 |
|  | 2 | 0.0 | 0.0 | 0.0 | 0.002 ±0.003 | 0.0 | 0.0 |
|  | 4 | 0.0 | 0.0 | 0.0 | 0.0 | 0.0 | 0.0 |
| **Propionic acid** | 1 | 0.0 | 0.0 | 0.075 ± 0.067 | 0.033 ± 0.057 | 0.030 ± 0.052 | 0.030 ± 0.052 |
|  | 2 | 0.0 | 0.0 | 0.030 ± 0.053 | 0.0 | 0.030 ± 0.052 | 0.0 |
|  | 4 | 0.0 | 0.032 ± 0.056 | 0.032 ± 0.056 | 0.0 | 0.092 ± 0.004 | 0.0 |
| **Butyric acid** | 1 | 0.0 | 0.0 | 0.0 | 0.002 ± 0.003 | 0.0 | 0.001 ± 0.002 |
|  | 2 | 0.0. | 0.0 | 0.0 | 0.002 ± 0.003 | 0.0 | 0.0 |
|  | 4 | 0.0 | 0.0. | 0.0 | 0.002 ± 0.003 | 0.0 | 0.0 |
| **Succinic acid** | 1 | 0.0 | 0.0 | 0.008 ± 0.014 | 0.0 | 0.009 ± 0.016 | 0.008 ± 0.013 |
|  | 2 | 0.0 | 0.0 | 0.009 ± 0.015 | 0.016 ± 0.028 | 0.008 ± 0.014 | 0.008 ± 0.014 |
|  | 4 | 0.0 | 0.0 | 0.0 | 0.016 ± 0.014 | 0.008 ± 0.014 | 0.009 ± 0.016 |

Table S2:The finall composition of the supernatants obtained over the course of PPR experiment S1, S2 and S3. Cycles 6 & 8 performed at lower carbon source concentration.

| **BES** | | **S1** | **S2** | **S3** |
| --- | --- | --- | --- | --- |
| Compound | Cycle | mM | mM | mM |
| Acetic acid | 2 | 0 | 0.323 ± 0.559 | 1.511 ± 2.158 |
|  | 4 | 0 | 0.799 ± 0.692 | 1.497 ± 1.529 |
|  | 6 | 0 | 0 | 0.250 ± 0.270 |
|  | 8 | 0 | 0 | 0.319 ± 0.322 |
| Ethanol | 2 | 0.455 ± 0.488 | 0.482 ± 0.489 | 0.270 ± 0.427 |
|  | 4 | 0.378 ± 0.140 | 0.264 ± 0.414 | 0.436 ± 0.060 |
|  | 6 | 0.333 ± 0.157 | 0.402 ± 0.141 | 0.067 ± 0.108 |
|  | 8 | 0.202 ± 0.350 | 0 | 0.224 ± 0.233 |
| Formic acid | 2 | 0 | 0 | 0.056 ± 0.097 |
|  | 4 | 0 | 0 | 0 |
|  | 6 | 0 | 0 | 0 |
|  | 8 | 0 | 0 | 0 |
| Propionic acid | 2 | 0 | 0 | 0 |
|  | 4 | 0 | 0 | 0 |
|  | 6 | 0.029 ± 0.050 | 0.029 ± 0.050 | 0.058 ± 0.002 |
|  | 8 | 0.029 ± 0.051 | 0.029 ± 0.050 | 0.089 ± 0.002 |
| Butyric acid | 2 | 0.005 ± 0.006 | 0.006 ± 0.001 | 0.010 ± 0.002 |
|  | 4 | 0 | 0 | 0 |
|  | 6 | 0 | 0 | 0 |
|  | 8 | 0 | 0 | 0 |
| Succinic acid | 2 | 0 | 0 | 0 |
|  | 4 | 0 | 0.008 ± 0.014 | 0 |
|  | 6 | 0 | 0.017 ± 0.015 | 0.008 ± 0.013 |
|  | 8 | 0.008 ± 0.014 | 0.016 ± 0.014 | 0.017 ± 0.015 |


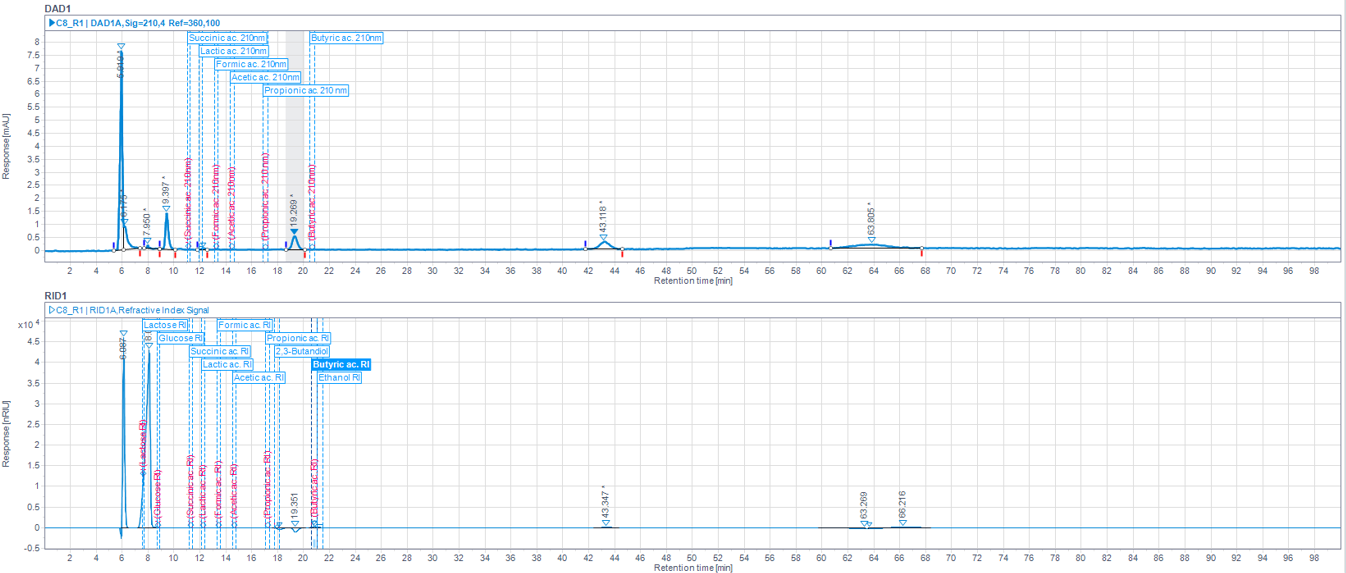


Figure S7: The chromatograms illustrating the composition of resulting supernatants after CA at -0.5 V during 1^st^ batch of HBPR for S1-BES.


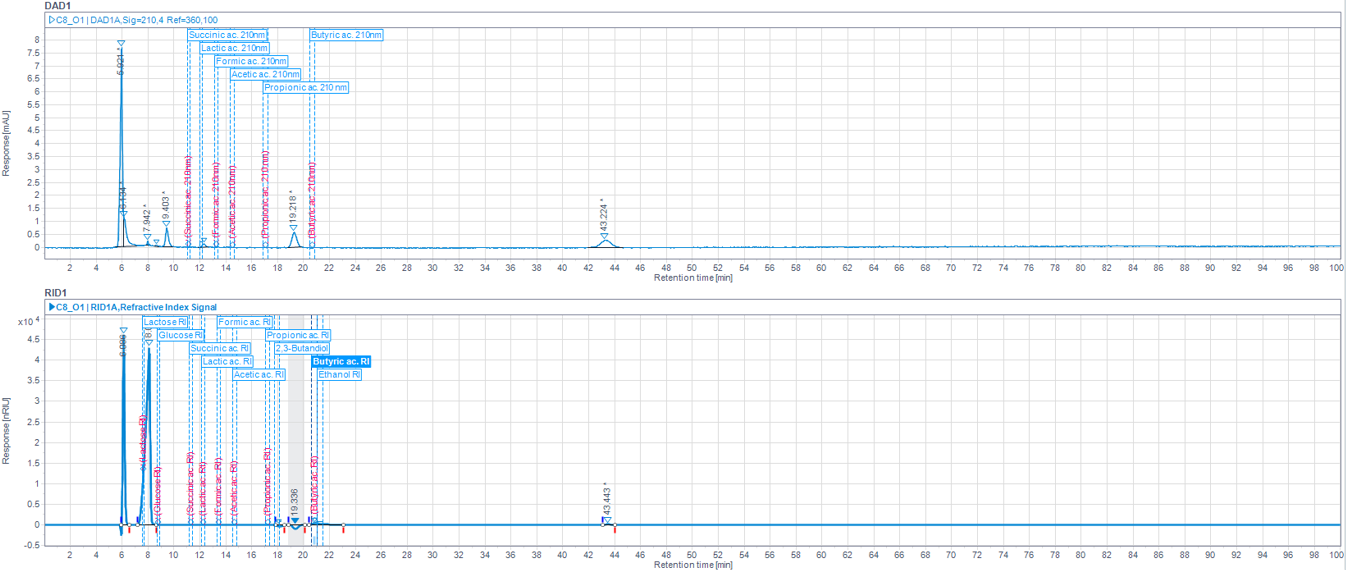


Figure S8: The chromatograms illustrating the composition of resulting supernatants after CA at +0.2 V during 1^st^ batch of HBPR for S1-BES.


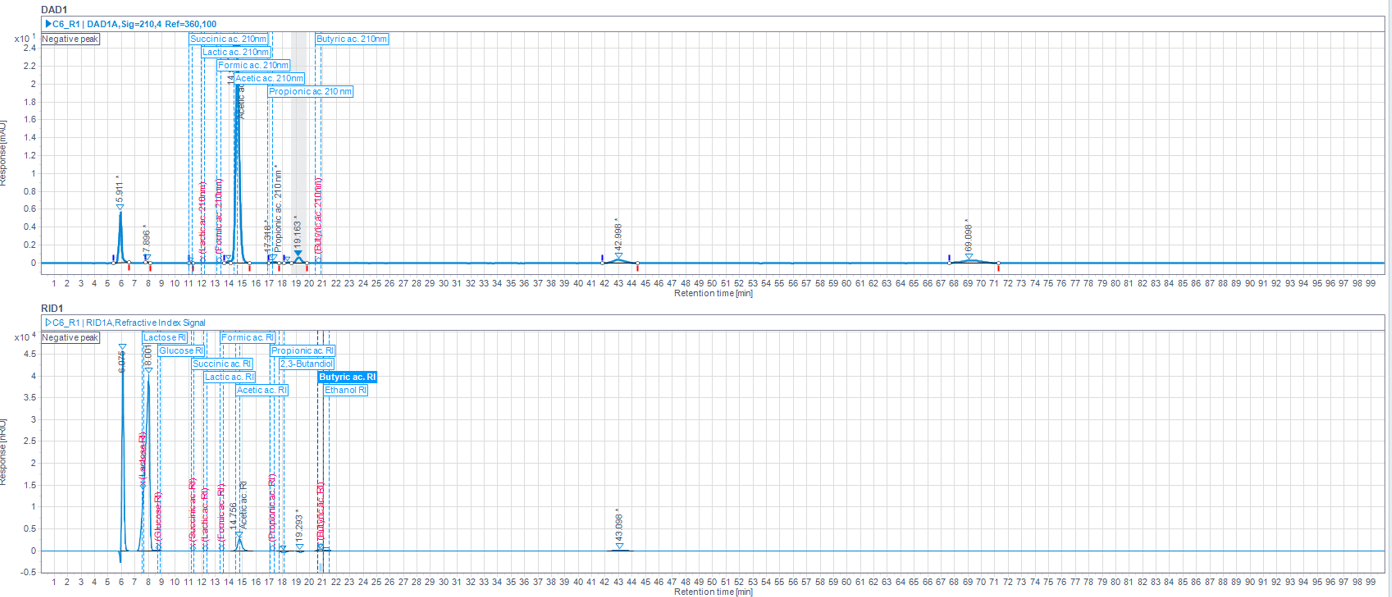


Figure S9: The chromatograms illustrating the composition of resulting supernatants after CA at -0.5 V during 1^st^ batch of HBPR for S2-BES.


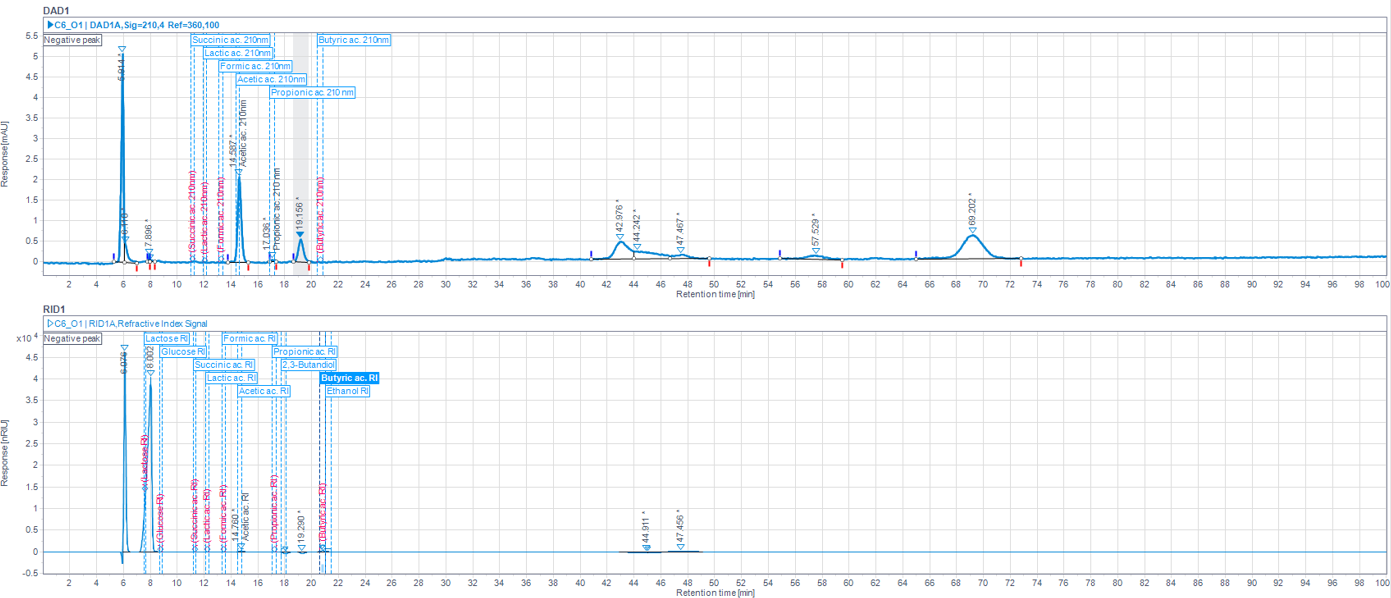


Figure S10: The chromatograms illustrating the composition of resulting supernatants after CA at +0.2 V during 1^st^ batch of HBPR for S2-BES.


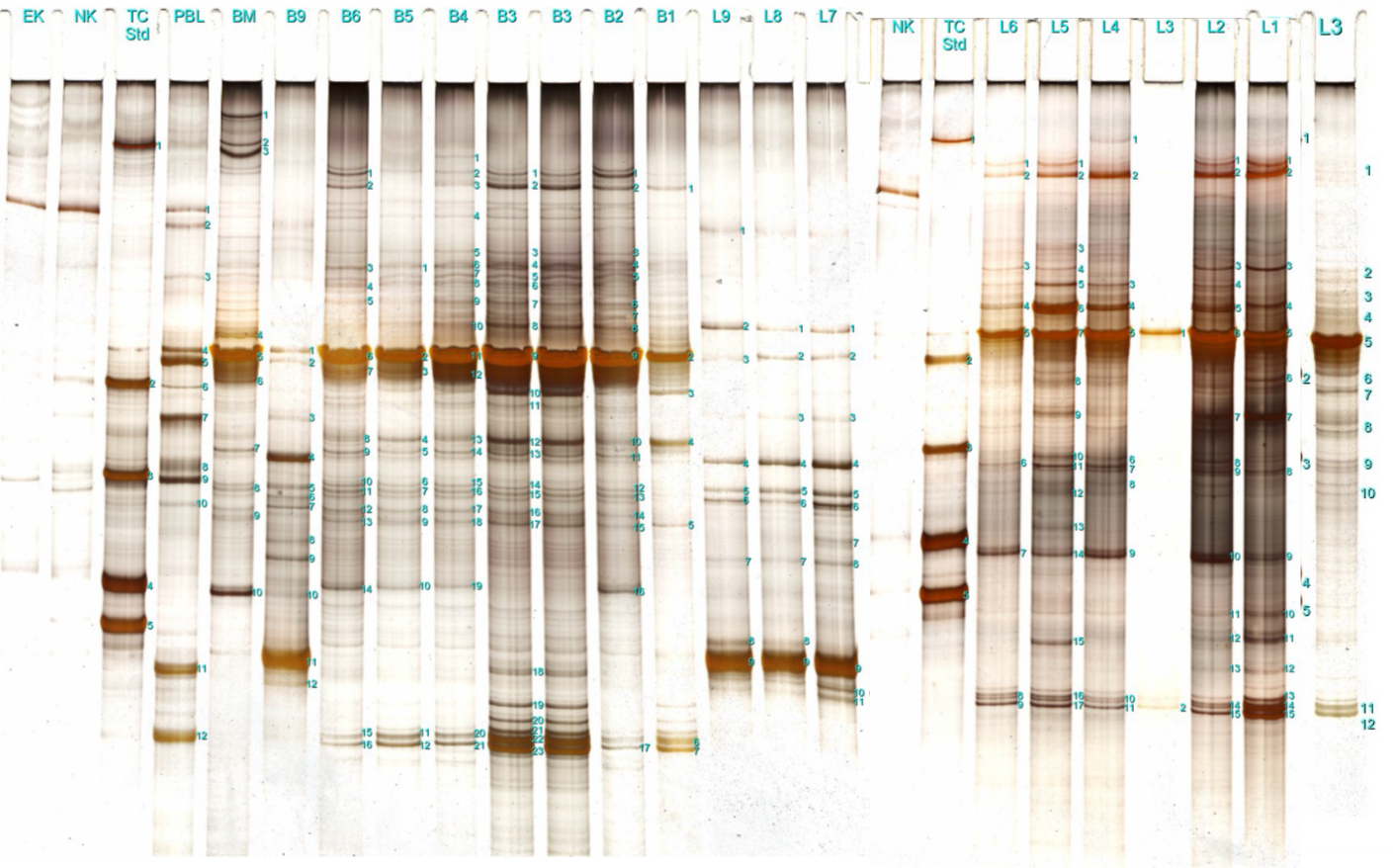


Figure S11: Two combined electrophoresis gel images after DNA staining, related to Figure 6. The gel images are aligned on last band (top) and scaled so that the lanes have the same width. PBL is the German abbreviation for PBS. The gel images were made by and provided by the German company Amodia Bioservice GmbH, who conducted the MCA.


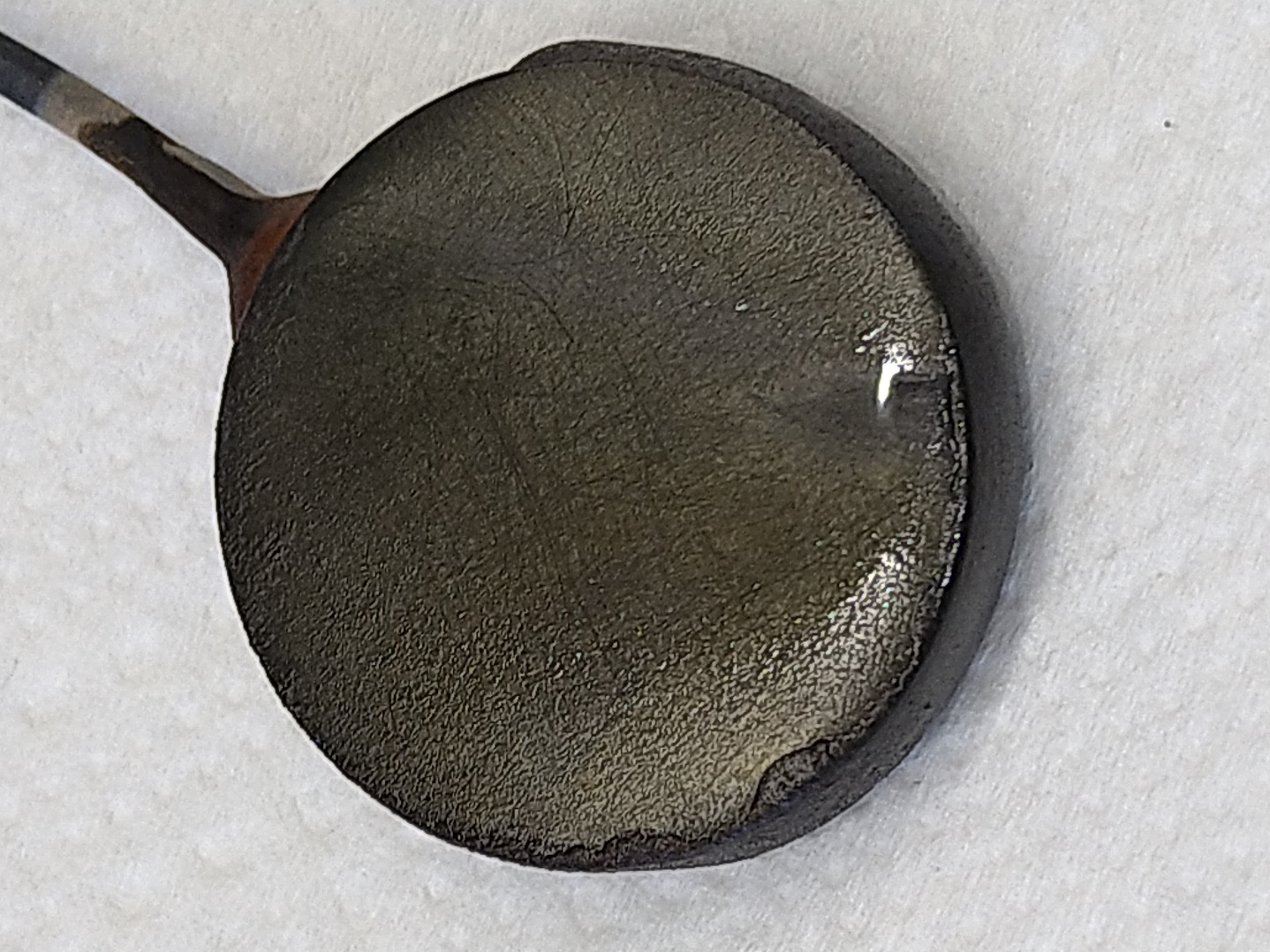


Figure S12: Only S1-BES, i.e., R9 which had biomass on the electrode. This is the B9 sample in Figure S-11.


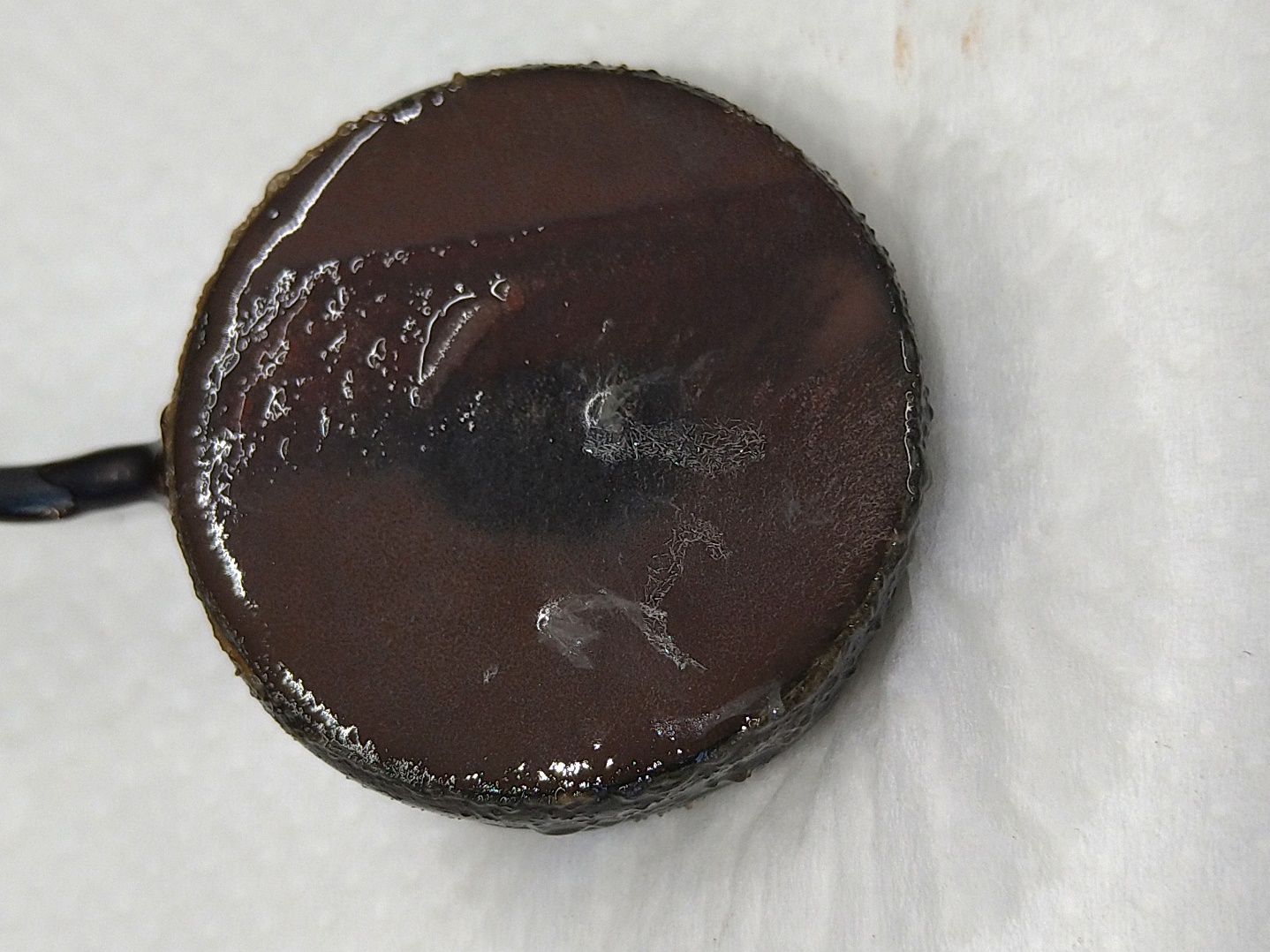


Figure S13: Electrode biofilm established in one of the S2-BES.


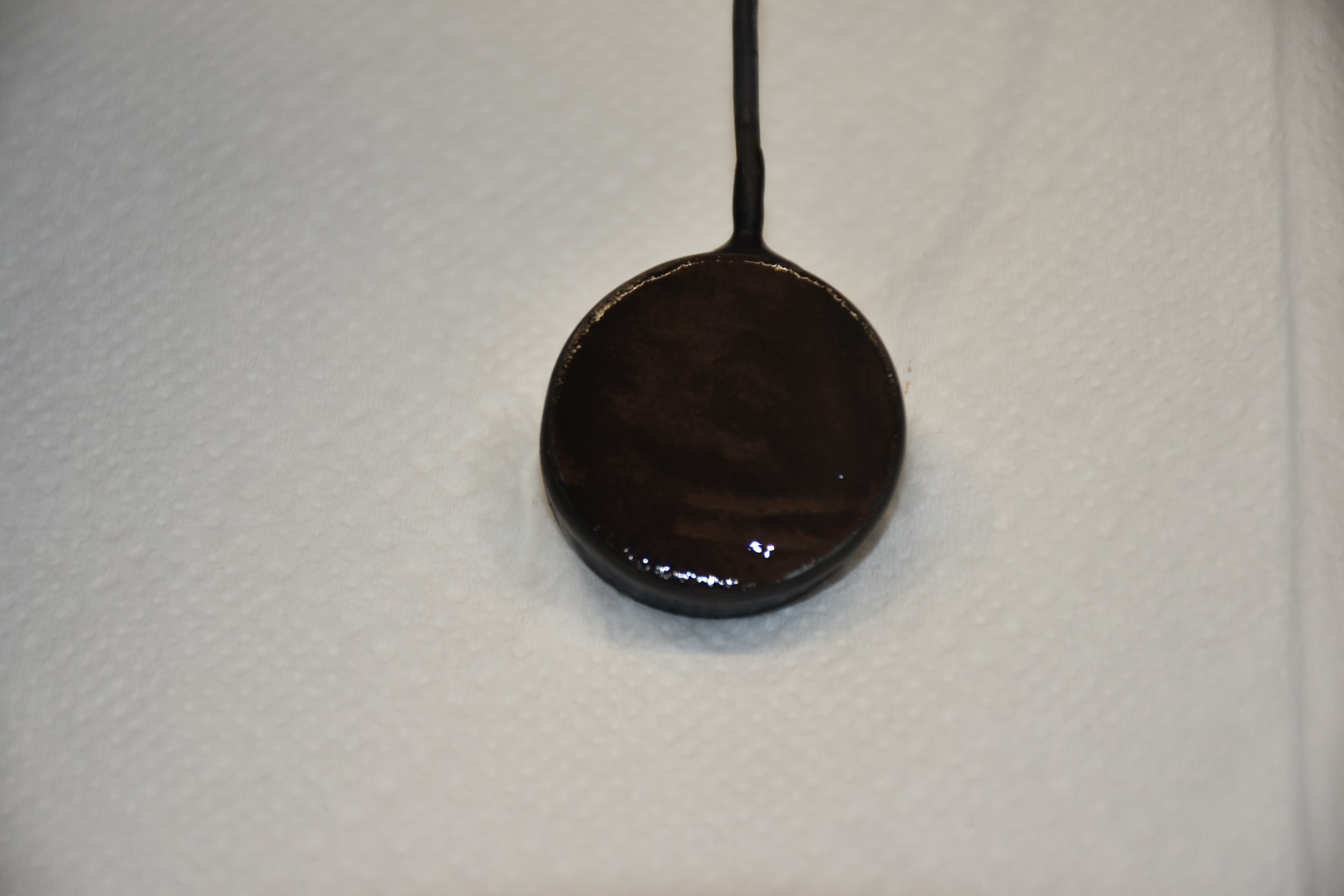


Figure S14: Electrode biofilm established in one of the S3-BES.

Figure S15: The relative abundance of the microbial communities in phosphate buffer solution (PBS) used for media preparation and BES-mother reactor biofilm biomass (BM) used as inoculum. n/s – no similarities.
